# Enhancement and Imputation of Peak Signal Enables Accurate Cell-Type Classification in scATAC-seq

**DOI:** 10.3389/fgene.2021.658352

**Published:** 2021-04-06

**Authors:** Zhe Cui, Ya Cui, Yan Gao, Tao Jiang, Tianyi Zang, Yadong Wang

**Affiliations:** ^1^Centre for Bioinformatics, School of Computer Science and Technology, Harbin Institute of Technology, Harbin, China; ^2^College of Life Science, University of Chinese Academy of Sciences, Beijing, China

**Keywords:** scATAC-seq, classification, machine learning, support vector machine, cell-type annotation

## Abstract

Single-cell Assay Transposase Accessible Chromatin sequencing (scATAC-seq) has been widely used in profiling genome-wide chromatin accessibility in thousands of individual cells. However, compared with single-cell RNA-seq, the peaks of scATAC-seq are much sparser due to the lower copy numbers (diploid in humans) and the inherent missing signals, which makes it more challenging to classify cell type based on specific expressed gene or other canonical markers. Here, we present svmATAC, a support vector machine (SVM)-based method for accurately identifying cell types in scATAC-seq datasets by enhancing peak signal strength and imputing signals through patterns of co-accessibility. We applied svmATAC to several scATAC-seq data from human immune cells, human hematopoietic system cells, and peripheral blood mononuclear cells. The benchmark results showed that svmATAC is free of literature-based markers and robust across datasets in different libraries and platforms. The source code of svmATAC is available at https://github.com/mrcuizhe/svmATAC under the MIT license.

## Introduction

With the technological progress in Single-cell Assay Transposase Accessible Chromatin sequencing(scATAC-seq) (Buenrostro et al., 2013), which has overcome the previous limitations and is able to generate thousands of single cells chromatin accessibility data at lower cost (Chen et al., 2019), a certain number of scATAC-seq datasets have been sequenced with different techniques in diverse libraries. For example, the Chromium Single Cell ATAC technology from 10X genomics (10X Genomic, 2020) can profile hundreds to tens of thousands of nuclei in one chip and finish the process from sample to sequencing-ready library in 1 day. For single-cell RNA-sequencing (scRNA-seq) and scATAC-seq data, the processing steps typically start with unsupervised clustering cells from coordinate-based peak matrix and then identify cell types from clustered groups. Thus, many methods requiring a training dataset labeled with corresponding cell populations for classifier training have been developed to get rid of the requirement of prior knowledge in scRNA-seq (Kiselev et al., 2018; Lieberman et al., 2018; Lopez et al., 2018; Boufea et al., 2019; Johnson et al., 2019; Ma and Pellegrini, 2019; Tan and Cahan, 2019). Support vector machine (SVM) performs the best among machine learning methods for classifying cell types in scRNA-seq (Abdelaal et al., 2019), and a lot of SVM-based tools have been proved effective and efficient (Pedregosa et al., 2011; Alquicira-Hernandez et al., 2019). However, the low copy number of DNA molecule in a cell results in only 1–10% of the accessible peaks in scATAC-seq being detectable, while the percentage for expressed genes detected in scRNA-seq is about 10–45% (Liu et al., 2019; Mereu et al., 2020). When clustering in scATAC-seq, such severe signal loss in a massive sparse space makes it more challenging to annotate cluster groups through gene-related canonical markers, which is practical and well-received in scRNA-seq. This missing of signal makes the SVM with linear kernel hard to work (Stewart et al., 2018) because this method starts with dimensionality reduction and feature selection, which is largely dependent on the accuracy and integrity of the dataset. Even so, SVM still outperformed other popular machine learning methods on cell-type classification of scATAC-seq (Cui et al., 2020), though the classification results of these methods (including SVM) are all performing at a low level. Since the signal missing will affect the quality of feature selection and then affects the construction of the classification model, the data recovery and signal strength enhancement are essential for SVM-based methods in scATAC-seq datasets (Yan et al., 2020).

Statistical methods such as imputing dropouts and correcting excess zero-counts have already been applied to scATAC-seq datasets, and this type of enhancing and recovering of missing signals has shown great power for downstream analysis. SCALE (Xiong et al., 2019) constructs a probabilistic Gaussian Mixture Model to characterize data, followed by denoising and imputing missing values in clustered subgroups. scOpen (Li et al., 2019) recovers the dropout signal in a particular cell using positive-unlabeled learning. However, these methods basically are using the statistic-based model, which may require an extra prior knowledge or time-consuming globally statistics. Since the repertoire of accessible regulatory elements in cell lines or tissues is unique, this type of data imputation is then considered as a kind of molecular signature for identifying. For example, Cicero (Pliner et al., 2018) is able to predictcis-regulatory DNA interactions through scATAC-seq from a single experiment.

**FIGURE 1:**
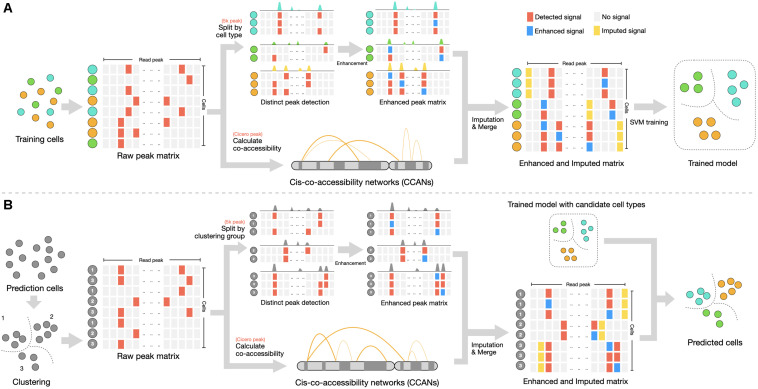
Summary of svmATAC method. **(A)** Training step. A fixed-size cell-peak matrix is constructed from a labeled scATAC-seq dataset. The peak matrix will be filtered and (1) matrix with 5 k peak will be split by cell types for calling distinct peak and signal in qualified peaks will be enhanced; (2) matrix with Cicero peak will be input to Cicero for calculating co-accessibility score between peaks. Two peaks with cis-regulatory interaction will be integrated for imputation in each enhanced peak matrix. All enhanced and imputed peak matrix will be merged to a single matrix for training SVM classifier. **(B)** Prediction step. A clustering is necessary at first for assigning each cell a clustering group number and then the matrix will be filtered, followed by enhancement and imputation steps. Finally, an SVM classifier will identify the cell types of predicting dataset using the SVM model trained from step A.

Here, we present svmATAC, an automatic cell classification SVM-based method for scATAC-seq data. svmATAC enhances the data from cluster/group data first, followed by imputing the signal linkage according to the co-accessibility scores from Cicero. The enhanced and imputed data will then be input to SVM (linear kernel) classifier for model training and cell-type prediction (Figure 1). We applied svmATAC to several typical scATAC-seq datasets containing different cell types, including human immune cell (hereafter Corces2016) (Corces et al., 2016), human hematopoietic system cell (hereafter Buenrostro et al., 2018), and peripheral blood mononuclear cell (hereafter 10 × PBMCs) (Genomic, 2020), to evaluate its classification ability. With fivefold cross-validation, svmATAC showed a great advance on prediction accuracy and surpassed 7.13–21.34% compared to SVM (linear kernel). In inter-dataset experiments, svmATAC also maintained great predictive power to accurately and quickly identify cell types based on a pre-trained model. We believe that svmATAC has great potential to handle complex cell-type identification problems in practical and realistic scenarios.

## Results

### svmATAC as a General Framework for Classification of scATAC-seq

svmATAC applies two pivotal functions, i.e., group-based read signal enhancement and cis-regulatory relationship-based imputation to cell-peak matrix, followed by training model and predicting cell types using SVM classifier (Figure 1). With this specific design, the peak signals of scATAC-seq are strengthened and related by extra biological connections, which improves the feature selection in lower dimensional space. svmATAC consists of three main steps: (1) It applies a specific design enhancement method to establish cell-peak matrix. The peak value 0 will be set to 1 when the peak (column) signal rate is larger than prior knowledge cutoff in a cell-type/cluster group. This step is able to correctly classify some of the cell types (Supplementary Tables 1–10), compared to directly using raw dataset, but it is still not good enough. (2)An imputation method, i.e., Cicero, is applied to construct the cis-regulatory relationship between peaks and to compute the co-accessibility scores. Two peaks of a cell-type/cluster group will be integrated for imputation when its co-accessibility score ≥ 0.25 (Pliner et al., 2018). That is, the value 1 will be assigned for both peaks if any one peak is distinct. (3) The cell-peak matrix processed by the two pivotal functions will be used as input for an SVM classifier to perform model training. With the trained SVM model, svmATAC can achieve the final prediction of cell types in unlabeled dataset. In order to give a comprehensive evaluation on the performance of svmATAC, we, respectively, designed an intra-dataset experiment and an inter-dataset experiment as below.

### Benchmark Results on Intra-Dataset Experiments

**FIGURE 2:**
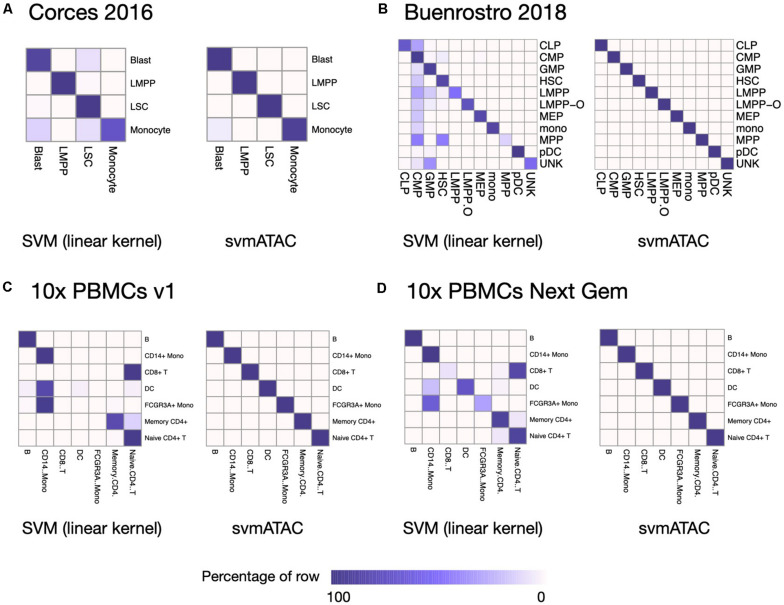
Heatmap comparing the SVM (linear kernel) and svmATAC predicted cells versus true label of intra-dataset experiment. **(A)** The experiment on Corces2016. In monocyte, the percentage of correctly predicted cells by svmATAC is increased the most, by 19.79% (from 75 to 94.79%), while the percentage of LSC is increased the least, by only 0.52% (from 98.96 to 99.48%), compared to SVM (linear kernel). **(B)** The experiment on Buenrostro2018. All cells are correctly classified by svmATAC, and the percentage of correctly predicted cells in all population increase by at most 86% in MPP, compared to the SVM (linear kernel). **(C)** The experiment on 10× PBMCs v1. All cells are correctly classified by svmATAC. The cells of CD8^+^ T and FCGR3A^+^ Mono, which are totally incorrectly classified by the SVM (linear kernel), are all correctly classified by svmATAC. **(D)** The experiment on 10× PBMCs Next Gem. All cells are correctly classified by svmATAC. The cells of CD8^+^ T, DC, and FCGR3A^+^ Mono, most of which are incorrectly classified by the SVM (linear kernel), are all correctly classified by svmATAC. Colors represent the percentages of cells of a specific reported type labeled as each type by svmATAC.

**FIGURE 3:**
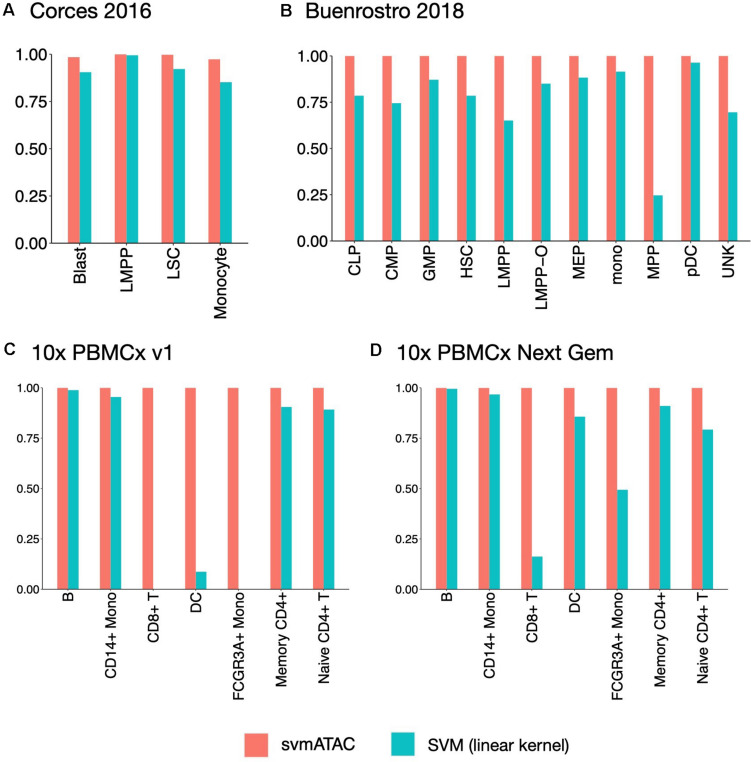
The F1 scores plot showing the performance comparison of SVM(linear kernel) and svmATAC for cell classification of intra-dataset experiment. **(A)** The experiment on Corces2016. svmATAC performed best on LMPP and its F1 score is 1 and the F1 score of monocyte increased the most, by 0.12 (from 0.85 to 0.97), compared to SVM (linear kernel). **(B)** The experiment on Buenrostro2018. The F1 scores of all cell types are 1 for svmATAC, which means that all cells are correctly classified and the F1 scores of all populations are increased by at most 0.75 (from 0.25 to 1) in MPP, compared to SVM (linear kernel). **(C)** The experiment on Seurat labeled 10× PBMCs v1. All cells are correctly classified by svmATAC and the F1 score of each cell type is 1. The F1 scores of CD8^+^ T and FCGR3A^+^ Mono, which are 0 when using SVM (linear kernel), are all increased to 1 for svmATAC. **(D)** The experiment on Seurat labeled 10× PBMCs Next Gem. All cells are correctly classified by svmATAC and the F1 score of each cell type is 1. The F1 scores of CD8^+^ T and FCGR3A^+^ Mono increased most by 0.84 (from 0.16 to 1) and 0.51 (from 0.49 to 1) when using SVM (linear kernel), compared to SVM (linear kernel). The red panel represents the results for svmATAC, and the blue panel represents the results for SVM (linear kernel) on unenhanced and unimputed data.

We evaluated the performance of svmATAC in an intra-dataset experiment by applying a fivefold cross-validation across each dataset after cell filtering. We randomly divided all the cells into fivefold with equal proportions of each cell population in each fold. The first and smallest dataset we used is from the human immune cells (hereafter Corces2016). This dataset consists of 576 immune cells from four isolated cell populations including leukemic blasts (Blast), lymphoid-primed multipotent progenitors (LMPP), leukemia stem cells (LSC), and monocytes. The gold standard labels we used here are from the original paper and predicted by enhancer cytometry. Compared to the SVM (linear kernel), we found an improvement on the predicted results when using svmATAC. The percentage of correctly predicted cells in all populations are all increased by at most 19.79% (from 75 to 94.79%) in monocyte (Figure 2A); the F1 scores are also improved in all population with monocyte increased the most from 0.85 to 0.97 (Figure 3A). The details for confusion matrix and F1 score list for Corces2016 are presented in Supplementary Tables 1, 2.

The second dataset we used is from the human hematopoietic system, which consists of 2,034 labeled hematopoietic cells from 10 cell populations including hematopoietic stem cells (HSC), multipotent progenitors (MPP), lymphoid-primed multipotent progenitors (LMPP), common myeloid progenitors (CMP), granulocyte-macrophage progenitors (GMP), GMP-like cells, megakaryocyte-erythroid progenitors (MEP), common lymphoid progenitors (CLP), monocytes (mono), and plasmacytoid dendritic cells (pDC). In order to test the ability of identifying the cells from different batches, we divided the LMPPs into two groups: LMPP-O: generated and first published in Corces2016; LMPP: newly generated and first published in Buenrostro2018. We used the FACS-sorting labels as the gold standard for this dataset. All cells in this dataset are correctly classified using svmATAC. Similar to the results on Corces2016, the percentage of correctly predicted cells in all population are increased by at most 86% in MPP (Figure 2B), and the F1 scores are also improved in all populations, with MPP increased the most from 0.25 to 1 (Figure 3B), compared to SVM (linear kernel). The details for confusion matrix and F1 score list for Buenrostro2018 are presented in Supplementary Tables 3, 4.

The last two datasets we used are from the peripheral blood mononuclear cells. These two datasets were generated from the same healthy donor but prepared in different libraries. In total, there are 3,917 cells profiled in 10× PBMCs v1 dataset and 4,585 cells were profiled in 10× PBMCs Next Gem dataset but both datasets are unlabeled. Based on recent studies (Bravo González-Blas et al., 2019; Pliner et al., 2019), we expected eight populations in each dataset, so we clustered cells into eight groups and use these cluster IDs as the gold label for training and testing (Supplementary Figures 1, 2). However, though cells with the same cluster ID may be predicted together into one group, we cannot check whether these predicted cell-types are true positives when only cluster ID is available. Thus, we assigned cell types using Seurat v3 (Stuart et al., 2019) based on a labeled scRNA-seq dataset from the same sample and then selected the high-confidence labels as gold standard for scATAC-seq datasets. We totally labeled 2,927 cells for the 10× PBMCs v1 dataset and 3,670 cells for the 10× PBMCs Next Gem dataset. For the Seurat labeled 10× PBMCs v1 dataset, the percentage of correctly predicted cells in each population increases to 100%, while CD8^+^ T, DC, and FCGR3A^+^ Mono are barely correctly identified at first using SVM (linear kernel) (Figure 2C); the F1 scores also improved in all populations, notably from 0 to 1 in CD8^+^, 0.09–1 in DC, and 0–1 in FCGR3A^+^ Mono (Figure 3C), compared to SVM (linear kernel). For the Seurat labeled 10× PBMCs Next Gem dataset, the percentage of correctly predicted cells in all population increases by at most 91% in CD8^+^ T (Figure 2D); the F1 scores also improved and CD8^+^ T increased the most from 0.16 to 1 (Figure 3D). The details for confusion matrix and F1 score list for the Seurat labeled 10× PBMCs v1 dataset and the Seurat labeled 10 × PBMCs Next Gem dataset are presented in Supplementary Tables 5–8.

### Benchmark Results on Inter-Dataset Experiments

In order to evaluate the ability of svmATAC to control or even overcome the deviation between different datasets such as batch effect, tissue type, and other technical factors, we designed the inter-dataset experiment, in which two datasets are generated from the same tissue, but prepared in different libraries and sequenced from different platforms.

We used Seurat labeled 10× PBMCs v1 to train a model first and then classify the labels of 10× PBMCs Next Gem based on this model. We compared the predicted labels with Seurat labels to evaluate the performance of svmATAC, and we found that although the model of the v1 dataset was trained on sparser molecular data from a different method and instrument, svmATAC is robust, performing well across datasets, and capable of overcoming batch effect and technical bias.

**FIGURE 4:**
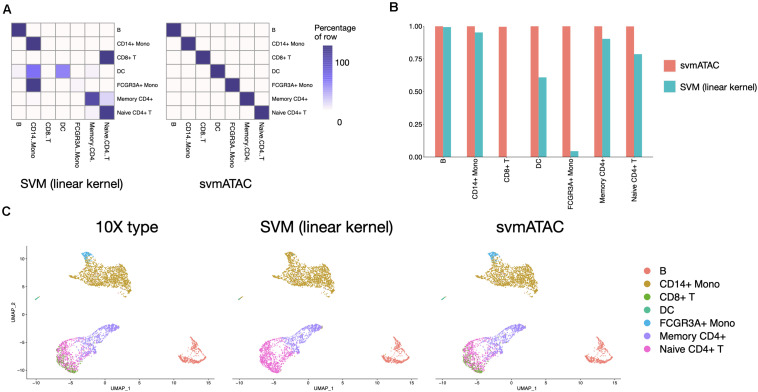
Summary of inter-dataset experiment. **(A)** Heatmap comparing the SVM (linear kernel) and svmATAC predicted cells versus true label of inter-dataset experiment. The cells of CD8^+^T and FCGR3A^+^ Mono are barely correctly classified when using SVM (linear kernel). svmATAC not only successfully classified all cells of CD8^+^T and FCGR3A^+^ Mono but also correctly classified all cells of other cell types, which increased the percentage of correctly predicted cells of all cell population from 47.96 to 99.95%. **(B)** The F1 scores plot displaying the performance comparison of SVM (linear kernel) and svmATAC for cell classification of inter-dataset experiment. All cells are correctly classified by svmATAC, and the F1 score of each cell type is 1. The F1 scores of CD8^+^ T and FCGR3A^+^ Mono, which are 0 when using SVM (linear kernel), are all increased to 1 in svmATAC. **(C)** Umap plots of the 10× PBMCs Next Gem (*n* = 3,670 cells). The first panel is colored by the true ground cell types of dataset, i.e., Seurat labeled cell types. The second panel is colored by the SVM (linear kernel) classification and the third panel is colored by the svmATAC classification.

svmATAC accurately classified 99.95% (3,668 out of 3,670) cells in the 10× PBMCs Next Gem dataset (Supplementary Tables 9, 10), compared to 47.96% using SVM (linear kernel) (Figure 4A). We also notice that all cells in the 10× PBMCs Next Gem dataset are correctly classified by svmATAC, even though the cells of CD8^+^ T and FCGR3A^+^ Mono are barely correctly classified when using SVM (linear kernel). Therefore, the F1 scores for all populations in svmATAC are all improved and CD8^+^ T and FCGR3A^+^ Mono increase the most by 0.996 (from 0 to 0.996) and 0.96 (from 0.04 to 1), respectively (Figure 4B).

We next investigated qualitatively the obtained classification results, using the respective feature matrices to project the cells onto a 2-D space using UMAP (McInnes et al., 2018) and colored them based on the obtained classification results or the gold standard labels. We found a high distribution consistency between true labels and svmATAC classified labels (Figure 4C), while SVM (linear kernel) misclassified most of the cells into two similar cell groups. Because of the close spatial distribution in lower-dimensional feature space, SVM (linear kernel) misclassified almost all cells of FCGR3A^+^ Mono and CD8^+^ T to CD14^+^ Mono and Naive CD4^+^ T, respectively. svmATAC not only successfully classified the almost all cells of these two cell types but also correctly classified all cells of other cell types.

## Discussion

Single-cell ATAC sequencing is a new technology in the area of the chromatin accessibility profile of individual cells and gives a new perspective of the identification and characterization of cell types (Cusanovich et al., 2015). Here, we introduced svmATAC, a specially designed method for scATAC-seq data to classify single cells based on readout enhancement, imputation, and a SVM model. The benchmark results show that svmATAC is able to accurately classify cells in both intra- and inter-datasets. The outstanding achievements of svmATAC are mainly due to its two pivotal modules: (1) the peak signal enhancement can overcome the disadvantage of read loss by sequencing technology; (2) the biological cis-regulatory relationship-based imputation can establish connections between significant regions.

However, there are still a few shortcomings for svmATAC that cannot be ignored. (1) In the current version of svmATAC, the accuracy and sensitivity of cell-type classification are highly relying on the manually selected cutoff for enhancement and imputation, which does exist a gap for applying svmATAC to more complex scATAC-seq datasets. We will develop an automatic cutoff adjustment for svmATAC in the future. (2) We also notice that a certain number of noisy read signals are added by mistake to the enhancement and imputation processes and decreases the performance especially in the inter-dataset experiment. This is another point for future work about how to avoid adding useless signal in enhancement and imputation steps. (3) Although svmATAC shows its potential on overcoming the batch effect on inter-dataset experiments using 10× datasets, we still expect more datasets coming from the same tissue or sample but generated through different sequencing pipelines.

Moreover, svmATAC also supports the user-defined classification model from all kinds of machine learning algorithms, which has great potentials in the adaptability in various scATAC-seq datasets. Therefore, svmATAC is a promising approach and benefits cutting-edge genomic studies.

## Materials and Methods

### Construction of Cell-Peak Matrix

Several region definitions for cell-peak matrix have been broadly used (Chen et al., 2019), including peaks on bulk data or aggregate single-cell data, pseudo-bulk data, regions around insertion sites, and fixed-size bins. The regions from bulk or aggregate scATAC-seq data are based on peak calling, and this process only keeps those areas covered by at least one read. The pseudo-bulk clades created by hierarchical clustering is different in the way of calling peaks, but the peaks are still generated from sequencing data. These regions around insertion sites do not rely on calling peaks from sequencing reads; however, this kind of peak region still only covers a part of the whole genome reference. These types of regions selection may be suitable for the developer’s application scenarios, such as clustering the cells into groups but cannot fulfill the requirement of svmATAC. This is because one of the most common scenarios for svmATAC is to predict the cell types for a dataset using a pre-trained classifier, which requires the two datasets used in training and predicting to share the same peak regions to ensure the compatibility of selected features.

We generated two types of cell-peak matrix containing different peak regions. One peak region is applying fixed-size peak regions (hereafter 5 k peak) for the training and predicting of the classifier process, in which we detected the read signal every 5,000 bp and therefore split the whole genome reference(hg19) into more than 600 k pieces. Note that some other tools may filter out the peaks with no read signal detected for saving memory and computing time, and we kept all the peaks here to make sure all regions for training data and predicting data are the same for compatibility of data structure. The other peak region is designed for Cicero; it is because we found that Cicero cannot process matrix with large regions spanning too large, such as 5 k here. We obtained a much smaller peak region (hereafter Cicero peak) from published data or bulk ATAC-seq data for matrix construction. This data matrix is only used for computing the co-accessibility score in the imputation process.

For the Coces2106 dataset, we first downloaded it from the NCBI database (GSE74310) and aligned it to hg19 using BWA-MEM (version 0.7.17-r1188) (Li, 2013) and enabled Picard (Broad_Institute, 2019) and Samtools (version 1.9) (Li et al., 2009) to remove the duplicated reads. Only duplication remove is applied to 10× PBMCs v1 and 10× PBMCs Next Gem dataset because these datasets are obtained in bam format from https://support.10xgenomics.com/single-cell-atac/datasets/1.2.0/atac_pbmc_5k_v1 and https://support.10xgenomics.com/single-cell-atac/datasets/1.2.0/atac_pbmc_5k_nextgem, respectively. These two 10× PBMCs datasets are downloaded with only cluster group ID available, but no true cell label was provided; we assigned labels to each cell by Seurat v3 as it can convincingly assign labels for scATAC-seq data when its scRNA-seq and labels are available. The peak and count file of Buenrostro2018 is available at GSE96769, and we obtained the aligned data from https://github.com/pinellolab/scATAC-benchmarking/tree/master/Real_Data/Buenrostro_2018.

Based on aligned and duplication-removed data and the cell labels provided in the datasets, we then estimated read coverage for each peak to build a cell-peak binary count matrix, in which each value 1 or value 0 represents whether a read signal was detected from the cell in this bin (1) or not (0). There is no limit to the number of cell types or the number of cells. Peaks that overlap ENCODE-defined blacklist regions are all set to zero. Cell populations with a size smaller than 10 were filtered. Note that for both kinds of peak region (5 k or Cicero), we did not filter out columns when all values are 0, which could be a kind of feature of classifier training.

Each cell matrix is represented in a compressed, sparse, column-oriented numeric matrix (dgCMatrix class in R). All these matrices are stored in RDS files and publicly available at https://github.com/mrcuizhe/svmATAC.

### Signal Strength Enhancement

The massive loss of read signal in scATAC-seq leads to incorrect zero counts of the cell-peak matrix, which may influence the training and prediction of the SVM classifier. Recovering the loss signal in data is a popular and workable way to strengthen the classification ability of machine learning classifiers, and this method has already been broadly accepted and developed in scRNA-seq data analysis, whose loss rate is a quarter less than that of scATAC-seq.

The enhancement process in svmATAC is trying to recover the inherent loss signal caused by sequencing techniques or experimental bias and then enhance the peak signal strength of each group. The enhancement procedure is a group-based step, in which data must be first divided into several groups based on its cell labels or clustering group numbers.

We first separated the cell-peak matrix by cell types into an*nm*matrix by cell types, i.e., a data matrix with *n*cells and *m*peaks, Then, we enhanced the read signal by recovering the missing signal using the following formula:

• When the fraction of non-zero cells of the *i*_*th*_ peak is larger than the cutoff for enhancement (i.e., $c_{e\,n\,h}\leq \frac{\sum_{j=1}^{n}C_{i,j}}{n}\leq 1$), we will treat all counts in the *i*_*th*_ peakas follows:

(1) $$S_{i}=\left[C_{i,1},\ldots ,C_{i,j},\ldots ,C_{i,n}\right],C_{i,j}=1$$

where *S_i* represents the read count for the *i*_*th*_ column (peak) in cell-peak matrix, *C*_*i,j*_ represents the read count of the *j*_*th*_ cell in the*i*_*th*_ column in matrix and i ∈ [1,*m*],*j* ∈ [1,*n*]. *c*_*enh*_ represents the cutoff for enhancement, and we recommend 0.1 here based on the read loss rate of scATAC-seq (Mereu et al., 2020; Liu et al., 2019) and experiment results (Supplementary Tables 1–10), which also shows that the enhancement step is efficient and necessary on scATAC-seq data for cell-type classification.

• When the percentage of non-zero cells of a peak is less than the cutoff for enhancement (i.e., 0≤$\frac{\sum_{j=1}^{n}C_{i,j}}{n}\leq c_{e\,n\,h}$), we will not change *S_i* and keep it intact.

### Signal Imputation

Apart from the enhancement of read signal, another way frequently applied in scATAC-seq data analysis is imputing read signal based on iconic biomarkers or biologic relationships, which may benefit the selection of features for each cell type. The imputation in svmATAC is also group-based and includes two steps:

• Compute the co-accessibility score for every two peaks.
  Co-accessibility scores represent the patterns and linkages of co-accessible pairs of DNA elements, such as distal elements and promoters. We use Cicero (v3.11, with default parameters) here to compute the co-accessibility scores for every two peaks. The co-accessibility score of each two peaks ranges from 0 to 1, indicating the strength of Cicero co-accessibility links. Scores closer to 1 indicate that two elements (peaks) are more co-accessible and vice versa.
• Imputing read signal based on cis-regulatory relationship into each group from co-accessibility score.
  Two peaks from enhanced data matrix will be considered as significantly connected if its co-accessibility score is higher than a threshold value. We first separated the enhanced cell-peak matrix by cell types into an*n* = *m* matrix, i.e., a data matrix with *n*cells and *m*peaks; then, all Cicero-linked peaks will be integrated for imputation using the following formula:
  When the Cicero co-accessibility score for the linkage between the *i*_*th*_ peak and *k*_*th*_ peak is higher than the cutoff for imputation and there is no zero cell for the *k*_*th*_ peak (i.e., $L_{i\,k}\geq c_{i\,n\,t}\,\text{and}\,\sum_{j=1}^{n}C_{k,j}=n$), we will treat all counts in the *i*_*th*_ peak as follows:

(2) $$S_{i}=\left[C_{i,1},\ldots ,C_{i,j},\ldots ,C_{i,n}\right]\,C_{i,j}=1$$

where*S_i_* represents the *i*_*th*_ column (peak) in cell-peak matrix, *C*_*i,j*_ represents the read count of the *j*_*th*_ cell in the*i*_*th*_peak in matrix and i,k ∈ [1,*m*],*j* ∈ [1,*n*]. *c*_*int*_ represents the cutoff for co-accessibility score, and we recommend 0.25 here based on the prior knowledge from the Cicero paper and experiment results (Supplementary Tables 1–10), which also shows that the enhancement step is efficient and necessary on scATAC-seq data for cell-type classification. *L*_*ik*_ represents the Cicero co-accessibility score for the linkage between the *i*_*th*_peak and the *k*_*th*_ peak.
When either the Cicero co-accessibility score for the linkage between the*i_þ_* peak and *k*_*þ*_ peak is lower than the cutoff for imputation or there is more than one non-zero cell for the *k*_*th*_ peak (i.e., $L_{i\,j}<c_{i\,n\,t}\,\text{or}\,\sum_{j=1}^{n}C_{k,j}\neq n$), we will not change the count value in the*i*_*th*_ peak and keep *S_i*intact.

Note that the matrix for computing Cicero co-accessibility score is based on Cicero peaks, which is different from the 5 k peak used for enhanced data matrix. Only the first (leftmost) 5 k peak will be considered for imputation if a peak from Cicero peaks is overlapped with multiple 5 k peaks. All imputed matrixes should be merged back into one matrix by columns(peaks) for downstream training and predicting.

### Classifier Training and Predicting

The enhanced and imputed cell-peak matrix will be used as input for SVM to train a classifier, and the trained classifier will then be used to predict cell types in an unlabeled dataset. We totally designed two types of experiments including intra-dataset and inter-dataset for evaluating the performance and adjusting the parameters in svmATAC.

In intra-dataset experiments, we performed a fivefold cross-validation on four datasets, including Corces2016, Buenrostro2018, 10× PBMCs v1, and 10× PBMCs Next Gem, to evaluate the classification ability of svmATAC. The folds were divided in a stratified manner to keep equal proportions of each cell population in each fold. The training and testing folds were same for all methods.

To evaluate the performance of svmATAC in more realistic scenarios (batch effect, technical factors, etc.), we designed an inter-dataset experiment, in which we trained a classifier based on 10× PBMCs v1 dataset and used this classifier to predict the cells of 10× PBMCs Next Gem dataset. Note that for the predicting dataset, since there are no known labels before classification and our process of enhancement and imputation are both group-based, a clustering is recommended to assign the cells a group number for following enhancement and imputation.

### Performance Evaluation Metrics

In this paper, we evaluated and compared the performance of SVM (linear kernel) and svmATAC using the following two metrics:

For all datasets, we compared the F1scores across different cell types and evaluated the performance of each method using mean F1scores.

F1 score is defined as:

(3) $$F\,1=\frac{2\times P\,r\,e\,c\,i\,s\,i\,o\,n\times R\,e\,c\,a\,l\,l}{P\,r\,e\,c\,i\,s\,i\,o\,n+R\,e\,c\,a\,l\,l}$$

where Precision is defined as:

(4) $$P\,r\,e\,c\,i\,s\,i\,o\,n=\frac{T\,r\,u\,e\,P\,o\,s\,i\,t\,i\,v\,e\,s}{T\,r\,u\,e\,P\,o\,s\,i\,t\,i\,v\,e\,s+F\,a\,l\,s\,e\,P\,o\,s\,i\,t\,i\,v\,e\,s}$$

Similarly, Recall (or the ratio of TPs to total calls in the truth set) is defined as:

(5) $$R\,e\,c\,a\,l\,l=\frac{T\,r\,u\,e\,P\,o\,s\,i\,t\,i\,v\,e\,s}{T\,r\,u\,e\,P\,o\,s\,i\,t\,i\,v\,e\,s+F\,a\,l\,s\,e\,N\,e\,g\,a\,t\,i\,v\,e\,s}$$

We represented the percentage of cells of a specific reported type labeled as each type in a heatmap, which flatly and intuitively showed the confusion matrix and the percentage of correctly/incorrectly classified cells.

The percentage of cells of a specific reported type labeled as each type is defined as:

(6) $$P\,e\,r\,c\,e\,n\,t\,a\,g\,e_{c\,e\,l\,l\,\_\,t\,y\,p\,e_{i},c\,e\,l\,l\,\_\,t\,y\,p\,e_{j}}=\frac{N_{c\,e\,l\,l\,\_\,t\,y\,p\,e_{i},c\,e\,l\,l\,\_\,t\,y\,p\,e_{j}}}{N_{c\,e\,l\,l\,\_\,t\,y\,p\,e_{i}}}$$

where *Percentage*_*cell*_*type*_*i*_,*cell*_*type*_*j*__ represents the percentage of those *cell*_*type*_*i*_cells labeled as *cell*_*type*_*j*_, *N*_*cell_type_i,cell_type_j*_ represents the number of those *cell*_*type*_*i*_cells labeled as *cell*_*type*_*j*_, and *N*_*cell*_*type*_*i*__ represents the total number of*cell*_*type*_*i*_.

## Data Availability Statement

The original contributions presented in the study are included in the article/Supplementary Material, further inquiries can be directed to the corresponding authors.

## Author Contributions

ZC and YC conceived the project. ZC, TZ, and YW supervised the project. ZC developed svmATAC. YG and TJ provided valuable suggestions for experiments design. ZC, TZ, and YW wrote the manuscript. All authors read and approved the final manuscript.

## Conflict of Interest

The authors declare that the research was conducted in the absence of any commercial or financial relationships that could be construed as a potential conflict of interest.

## Supplementary Material

Supplementary Figure 1 Heatmap and F1-score comparing the SVM (linear kernel) and svmATAC predicted cells cluster versus original cluster in intra-dataset experiment. **(A)** Heatmap displaying the confusion matrix of predicted cell cluster ID versus original cluster ID in 10× PBMCs v1 with cluster ID dataset. **(B)** Heatmap displaying the confusion matrix of predicted cell cluster ID versus original cluster ID in 10× PBMCs Next Gem with cluster ID dataset. **(C)** Bar plot displaying the f1 scores of 10× PBMCs v1 with cluster ID. **(D)** Bar plot displaying the f1 score of 10× PBMCs Next Gem with cluster ID. Colors of **(A,B)** represent the percentages of cells of a specific reported type labeled as each type by svmATAC. In **(C,D)**, the red panel represent the results for svmATAC, and blue panel represents the results for general SVM on unenhanced and unimputed data.

Supplementary Figure 2 Heatmap comparing the SVM (linear kernel) and svmATAC predicted cells cluster versus original cluster ID in inter-dataset experiment. **(A)** 10× PBMCs v1 with cluster ID dataset. **(B)** 10× PBMCs Next Gem with cluster ID dataset. Colors represent the percentages of cells of a specific reported type labeled as each type by svmATAC.

Supplementary Table 1 F1 scores of intra-dataset experiment using Corces2016 dataset with different enhancement and imputation cutoffs.

Supplementary Table 2 The confusion matrix across different enhancement and imputation cutoffs.

Supplementary Table 3 F1 scores of intra-dataset experiment using Buenrostro2018 dataset with different enhancement and imputation cutoffs.

Supplementary Table 4 The confusion matrix across different enhancement and imputation cutoffs for Buenrostro2018 dataset.

Supplementary Table 5 F1 scores of intra-dataset experiment using 10× PBMCs v1 Seurat Labeled dataset with different enhancement and imputation cutoffs.

Supplementary Table 6 The confusion matrix across different enhancement and imputation cutoffs for 10× PBMCs v1 Seurat Labeled dataset.

Supplementary Table 7 F1 scores of intra-dataset experiment using 10× PBMCs Next Gem Seurat Labeled dataset with different enhancement and imputation cutoffs.

Supplementary Table 8 The confusion matrix across different enhancement and imputation cutoffs for 10× PBMCs Next Gem Seurat Labeled dataset.

Supplementary Table 9 F1 scores of inter-dataset experiment that training with 10× PBMCs v1 Seurat Labeled dataset and predicting in 10× PBMCs Next Gem Seurat Labeled dataset with different enhancement and imputation cutoffs.

Supplementary Table 10 The confusion matrix across different enhancement and imputation cutoffs for inter-dataset experiment that training with 10× PBMCs v1 Seurat Labeled dataset and predicting in 10× PBMCs Next Gem Seurat Labeled dataset.
