# Supplementary figures and images for: Enhancement and Imputation of Peak Signal Enables Accurate Cell-Type Classification in scATAC-seq
Source: Front Genet. 2021 Apr 6;12:658352. doi: 10.3389/fgene.2021.658352 (PMC8056015; doi:10.3389/fgene.2021.658352)

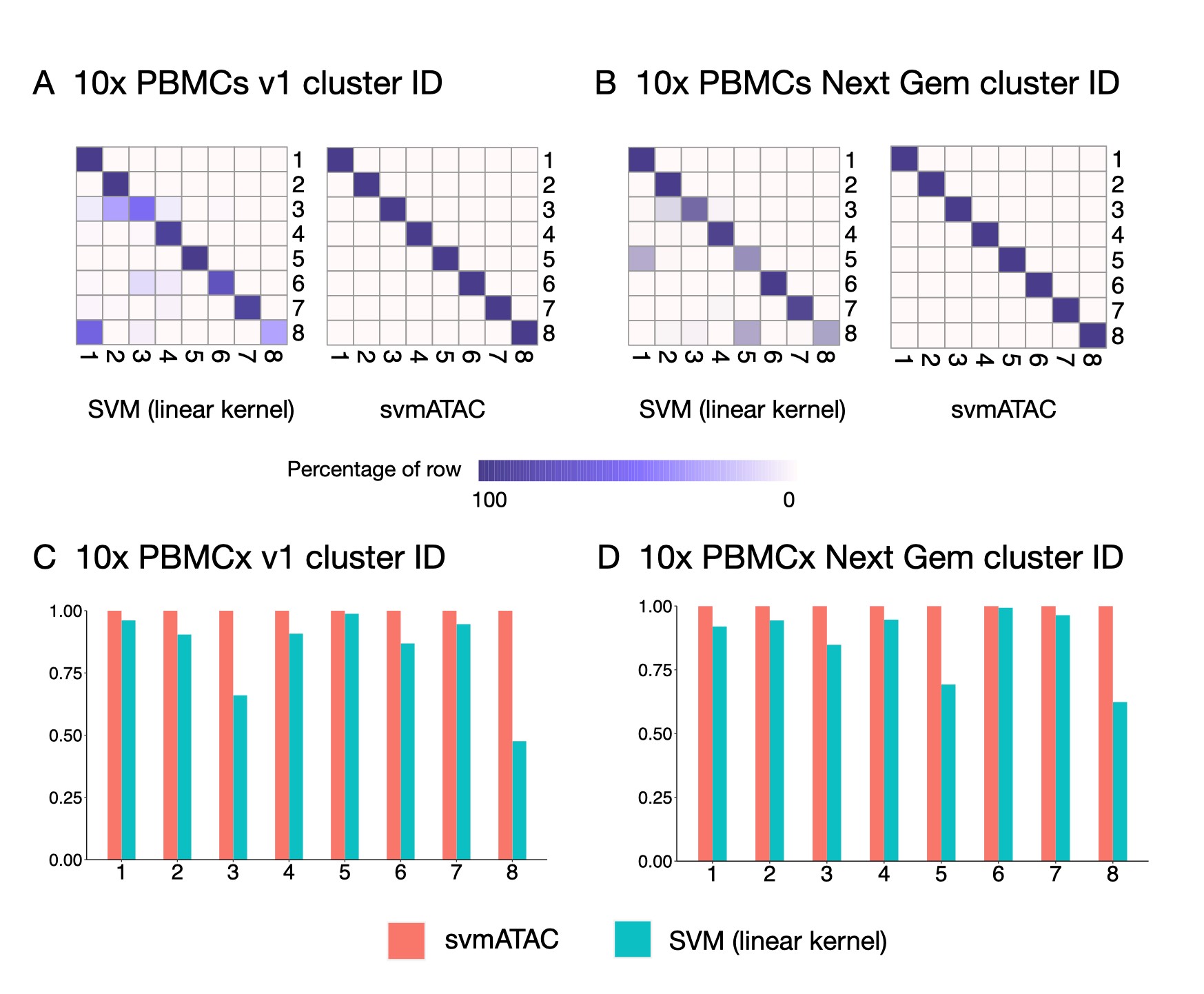

Supplement: Supplementary Figure 1 — Heatmap and F1-score comparing the SVM (linear kernel) and svmATAC predicted cells cluster versus original cluster in intra-dataset experiment. (A) Heatmap displaying the confusion matrix of predicted cell cluster ID versus original cluster ID in 10× PBMCs v1 with cluster ID dataset. (B) Heatmap displaying the confusion matrix of predicted cell cluster ID versus original cluster ID in 10× PBMCs Next Gem with cluster ID dataset. (C) Bar plot displaying the f1 scores of 10× PBMCs v1 with cluster ID. (D) Bar plot displaying the f1 score of 10× PBMCs Next Gem with cluster ID. Colors of (A,B) represent the percentages of cells of a specific reported type labeled as each type by svmATAC. In (C,D), the red panel represent the results for svmATAC, and blue panel represents the results for general SVM on unenhanced and unimputed data. [file Image_1.JPEG]

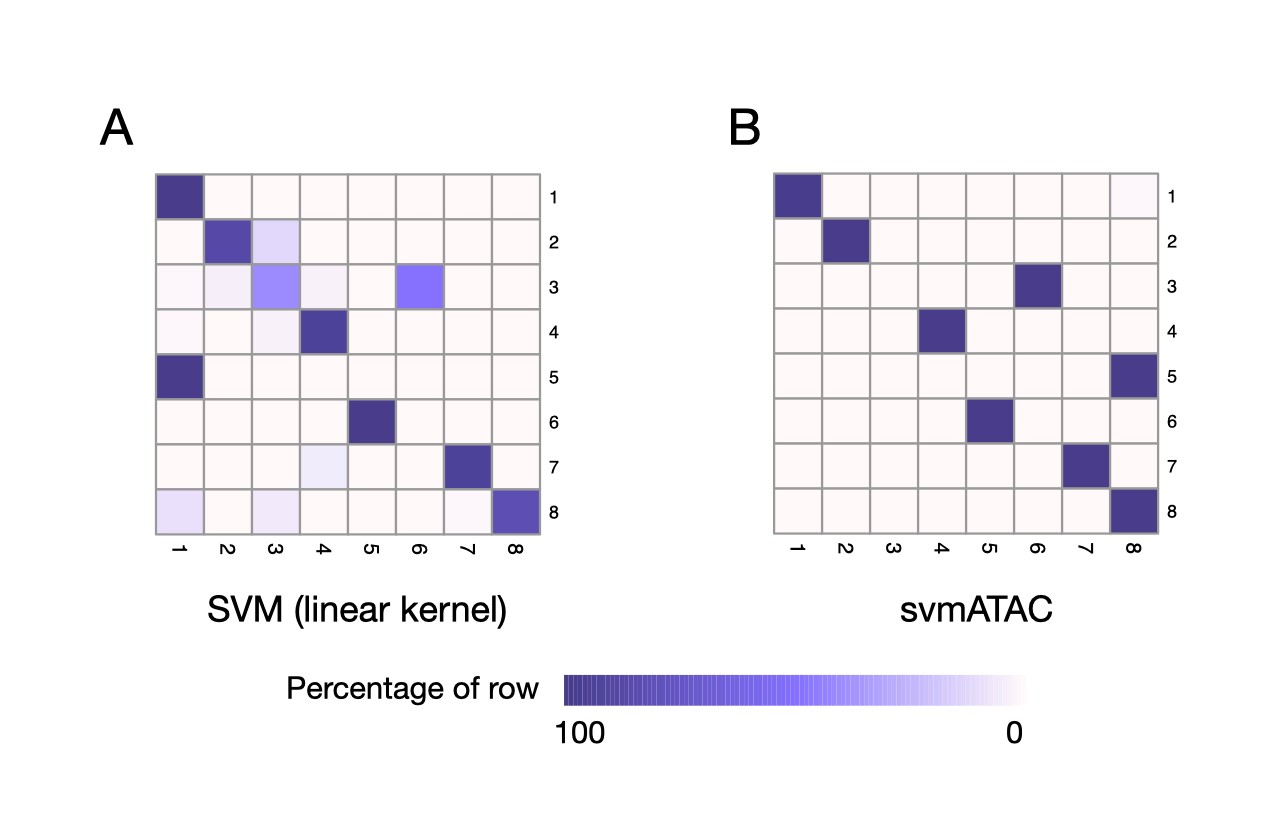

Supplement: Supplementary Figure 2 — Heatmap comparing the SVM (linear kernel) and svmATAC predicted cells cluster versus original cluster ID in inter-dataset experiment. (A) 10× PBMCs v1 with cluster ID dataset. (B) 10× PBMCs Next Gem with cluster ID dataset. Colors represent the percentages of cells of a specific reported type labeled as each type by svmATAC. [file Image_2.JPEG]
