# Supplementary material for: Enhancement and Imputation of Peak Signal Enables Accurate Cell-Type Classification in scATAC-seq
Source: Front Genet. 2021 Apr 6;12:658352. doi: 10.3389/fgene.2021.658352 (PMC8056015; doi:10.3389/fgene.2021.658352)
Supplement: Supplementary Table 1 — F1 scores of intra-dataset experiment using Corces2016 dataset with different enhancement and imputation cutoffs. [file Table_1.docx]

**Supplementary Table 1 F1 scores of intra-dataset experiment using Corces2016 dataset with different enhancement and imputation cutoffs**

| **F1 score** | **Blast** | **LMPP** | **LSC** | **Monocyte** |
| --- | --- | --- | --- | --- |
| No Enhancement & No Imputation | 0.9052632 | 0.9947644 | 0.9219512 | 0.852071 |
| Enh 0.3 & No Imp | 0.9052632 | 0.9947644 | 0.9219512 | 0.852071 |
| Enh 0.3 & Imp 0.75 | 0.9052632 | 0.9947644 | 0.9219512 | 0.852071 |
| Enh 0.3 & Imp 0.5 | 0.9052632 | 0.9947644 | 0.9219512 | 0.852071 |
| Enh 0.3 & Imp 0.25 | 0.9052632 | 0.9947644 | 0.9219512 | 0.852071 |
| Enh 0.2 & No Imp | 0.91777188 | 1 | 0.9473684 | 0.945055 |
| Enh 0.2 & Imp 0.75 | 0.91777188 | 1 | 0.9473684 | 0.945055 |
| Enh 0.2 & Imp 0.5 | 0.91777188 | 1 | 0.9473684 | 0.945055 |
| Enh 0.2 & Imp 0.25 | 0.91777188 | 1 | 0.9473684 | 0.945055 |
| Enh 0.1 & No Imp | 0.969697 | 1 | 0.9973753 | 0.9392265 |
| Enh 0.1 & Imp 0.75 | 0.9721519 | 1 | 0.9973753 | 0.945055 |
| Enh 0.1 & Imp 0.5 | 0.9770992 | 1 | 0.9973753 | 0.9565217 |
| Enh 0.1 & Imp 0.25 | 0.9846154 | 1 | 0.9973753 | 0.973262 |
