# Supplementary material for: Enhancement and Imputation of Peak Signal Enables Accurate Cell-Type Classification in scATAC-seq
Source: Front Genet. 2021 Apr 6;12:658352. doi: 10.3389/fgene.2021.658352 (PMC8056015; doi:10.3389/fgene.2021.658352)
Supplement: Supplementary Table 2 — The confusion matrix across different enhancement and imputation cutoffs. [file Table_2.DOCX]

**Supplementary Table 2 The confusion matrix across different enhancement and imputation cutoffs for Corces2016 dataset**

| **No Enh & No Imp** | | | | |
| --- | --- | --- | --- | --- |
|  | **Blast** | **LMPP** | **LSC** | **Monocyte** |
| **Blast** | 172 | 0 | 19 | 1 |
| **LMPP** | 0 | 95 | 1 | 0 |
| **LSC** | 2 | 0 | 189 | 0 |
| **Monocyte** | 14 | 0 | 10 | 72 |
| **Enh 0.3 & No Imp** | | | | |
|  | **Blast** | **LMPP** | **LSC** | **Monocyte** |
| **Blast** | 172 | 0 | 19 | 1 |
| **LMPP** | 0 | 95 | 1 | 0 |
| **LSC** | 2 | 0 | 189 | 0 |
| **Monocyte** | 14 | 0 | 10 | 72 |
| **Enh 0.2 & No Imp** | | | | |
|  | **Blast** | **LMPP** | **LSC** | **Monocyte** |
| **Blast** | 173 | 0 | 19 | 0 |
| **LMPP** | 0 | 96 | 0 | 0 |
| **LSC** | 2 | 0 | 189 | 0 |
| **Monocyte** | 10 | 0 | 0 | 86 |
| **Enh 0.1 & No Imp** | | | | |
|  | **Blast** | **LMPP** | **LSC** | **Monocyte** |
| **Blast** | 192 | 0 | 0 | 0 |
| **LMPP** | 0 | 96 | 0 | 0 |
| **LSC** | 1 | 0 | 190 | 0 |
| **Monocyte** | 11 | 0 | 0 | 85 |
| **Enh 0.3 & Imp 0.75** | | | | |
|  | **Blast** | **LMPP** | **LSC** | **Monocyte** |
| **Blast** | 172 | 0 | 19 | 1 |
| **LMPP** | 0 | 95 | 1 | 0 |
| **LSC** | 2 | 0 | 189 | 0 |
| **Monocyte** | 14 | 0 | 10 | 72 |
| **Enh 0.2 & Imp 0.75** | | | | |
|  | **Blast** | **LMPP** | **LSC** | **Monocyte** |
| **Blast** | 173 | 0 | 19 | 0 |
| **LMPP** | 0 | 96 | 0 | 0 |
| **LSC** | 2 | 0 | 189 | 0 |
| **Monocyte** | 10 | 0 | 0 | 86 |
| **Enh 0.1 & Imp 0.75** | | | | |
|  | **Blast** | **LMPP** | **LSC** | **Monocyte** |
| **Blast** | 192 | 0 | 0 | 0 |
| **LMPP** | 0 | 96 | 0 | 0 |
| **LSC** | 1 | 0 | 190 | 0 |
| **Monocyte** | 10 | 0 | 0 | 86 |
| **Enh 0.3 & Imp 0.5** | | | | |
|  | **Blast** | **LMPP** | **LSC** | **Monocyte** |
| **Blast** | 172 | 0 | 19 | 1 |
| **LMPP** | 0 | 95 | 1 | 0 |
| **LSC** | 2 | 0 | 189 | 0 |
| **Monocyte** | 14 | 0 | 10 | 72 |
| **Enh 0.2 & Imp 0.5** | | | | |
|  | **Blast** | **LMPP** | **LSC** | **Monocyte** |
| **Blast** | 173 | 0 | 19 | 0 |
| **LMPP** | 0 | 96 | 0 | 0 |
| **LSC** | 2 | 0 | 189 | 0 |
| **Monocyte** | 10 | 0 | 0 | 86 |
| **Enh 0.1 & Imp 0.5** | | | | |
|  | **Blast** | **LMPP** | **LSC** | **Monocyte** |
| **Blast** | 192 | 0 | 0 | 0 |
| **LMPP** | 0 | 96 | 0 | 0 |
| **LSC** | 1 | 0 | 190 | 0 |
| **Monocyte** | 8 | 0 | 0 | 88 |
| **Enh 0.3 & Imp 0.25** | | | | |
|  | **Blast** | **LMPP** | **LSC** | **Monocyte** |
| **Blast** | 172 | 0 | 19 | 1 |
| **LMPP** | 0 | 95 | 1 | 0 |
| **LSC** | 2 | 0 | 189 | 0 |
| **Monocyte** | 14 | 0 | 10 | 72 |
| **Enh 0.2 & Imp 0.25** | | | | |
|  | **Blast** | **LMPP** | **LSC** | **Monocyte** |
| **Blast** | 173 | 0 | 19 | 0 |
| **LMPP** | 0 | 96 | 0 | 0 |
| **LSC** | 2 | 0 | 189 | 0 |
| **Monocyte** | 10 | 0 | 0 | 86 |
| **Enh 0.1 & Imp 0.25** | | | | |
|  | **Blast** | **LMPP** | **LSC** | **Monocyte** |
| **Blast** | 192 | 0 | 0 | 0 |
| **LMPP** | 0 | 96 | 0 | 0 |
| **LSC** | 1 | 0 | 190 | 0 |
| **Monocyte** | 5 | 0 | 0 | 91 |

*Note*: In each table, the row represents the true label of cells and column represents the predicted label of cells
