# Supplementary material for: Enhancement and Imputation of Peak Signal Enables Accurate Cell-Type Classification in scATAC-seq
Source: Front Genet. 2021 Apr 6;12:658352. doi: 10.3389/fgene.2021.658352 (PMC8056015; doi:10.3389/fgene.2021.658352)
Supplement: Supplementary Table 3 — F1 scores of intra-dataset experiment using Buenrostro2018 dataset with different enhancement and imputation cutoffs. [file Table_3.DOCX]

**Supplementary Table 3 F1 scores of intra-dataset experiment using Buenrostro2018 dataset with different enhancement and imputation cutoffs**

| **F1 score** | **CLP** | **CMP** | **GMP** | **HSC** | **LMPP** | **LMPP-O** | **MEP** | **mono** | **MPP** | **pDC** | **UNK** |
| --- | --- | --- | --- | --- | --- | --- | --- | --- | --- | --- | --- |
| No Enhancement & No Imputation | 0.7851852 | 0.7447154 | 0.871481 | 0.785124 | 0.6511628 | 0.8503937 | 0.8828125 | 0.9152542 | 0.2469136 | 0.9637681 | 0.6956522 |
| Enh 0.3 & No Imp | 0.9873418 | 0.7881356 | 0.8793103 | 0.7867036 | 0.6717557 | 0.859375 | 1 | 0.9677419 | 0.2138365 | 0.9819495 | 0.8118812 |
| Enh 0.3 & Imp 0.75 | 0.9873418 | 0.7891617 | 0.8793103 | 0.7850208 | 0.6818182 | 0.859375 | 1 | 0.9677419 | 0.2138365 | 0.9782609 | 0.8118812 |
| Enh 0.3 & Imp 0.5 | 0.9936306 | 0.7868576 | 0.8765432 | 0.7844228 | 0.6511628 | 0.859375 | 0.9963636 | 0.9593496 | 0.2138365 | 0.967033 | 0.8888889 |
| Enh 0.3 & Imp 0.25 | 1 | 0.7898734 | 0.886392 | 0.7855153 | 0.640625 | 0.9264706 | 0.989011 | 0.9593496 | 0.2138365 | 0.9745455 | 0.9473684 |
| Enh 0.2 & No Imp | 0.9936306 | 0.806563 | 0.8942675 | 0.7837079 | 0.9824561 | 0.979021 | 0.9852941 | 0.9593496 | 0.2138365 | 0.967033 | 0.9565217 |
| Enh 0.2 & Imp 0.75 | 0.9936306 | 0.806563 | 0.8942675 | 0.7837079 | 0.9824561 | 0.979021 | 0.9852941 | 0.9593496 | 0.2138365 | 0.967033 | 0.9565217 |
| Enh 0.2 & Imp 0.5 | 1 | 0.806563 | 0.8965517 | 0.7837079 | 0.9883721 | 0.979021 | 0.9852941 | 0.9593496 | 0.2138365 | 0.9745455 | 0.9565217 |
| Enh 0.2 & Imp 0.25 | 1 | 0.8079242 | 0.9023136 | 0.7864215 | 0.9883721 | 1 | 0.9852941 | 0.9677419 | 0.2138365 | 0.9819495 | 0.9655172 |
| Enh 0.1 & No Imp | 1 | 1 | 1 | 1 | 1 | 1 | 1 | 1 | 1 | 1 | 1 |
| Enh 0.1 & Imp 0.75 | 1 | 1 | 1 | 1 | 1 | 1 | 1 | 1 | 1 | 1 | 1 |
| Enh 0.1 & Imp 0.5 | 1 | 1 | 1 | 1 | 1 | 1 | 1 | 1 | 1 | 1 | 1 |
| Enh 0.1 & Imp 0.25 | 1 | 1 | 1 | 1 | 1 | 1 | 1 | 1 | 1 | 1 | 1 |
