# Supplementary material for: Enhancement and Imputation of Peak Signal Enables Accurate Cell-Type Classification in scATAC-seq
Source: Front Genet. 2021 Apr 6;12:658352. doi: 10.3389/fgene.2021.658352 (PMC8056015; doi:10.3389/fgene.2021.658352)
Supplement: Supplementary Table 4 — The confusion matrix across different enhancement and imputation cutoffs for Buenrostro2018 dataset. [file Table_4.DOCX]

**Supplementary Table 4 The confusion matrix across different enhancement and imputation cutoffs for Buenrostro2018 dataset**

| **No Enh & No Imp** | | | | | | | | | | | | | | | | | | | | | | |
| --- | --- | --- | --- | --- | --- | --- | --- | --- | --- | --- | --- | --- | --- | --- | --- | --- | --- | --- | --- | --- | --- | --- |
|  | | **CLP** | **CMP** | | **GMP** | | **HSC** | | **LMPP** | | **LMPP-O** | | **MEP** | | **Mono** | | **MPP** | | **pDC** | | **UNK** | |
| **CLP** | | 53 | 24 | | 1 | | 0 | | 0 | | 0 | | 0 | | 0 | | 0 | | 0 | | 0 | |
| **CMP** | | 0 | 458 | | 12 | | 27 | | 0 | | 0 | | 5 | | 0 | | 0 | | 0 | | 0 | |
| **GMP** | | 3 | 41 | | 356 | | 0 | | 0 | | 0 | | 0 | | 0 | | 0 | | 2 | | 0 | |
| **HSC** | | 0 | 62 | | 0 | | 285 | | 0 | | 0 | | 0 | | 0 | | 0 | | 0 | | 0 | |
| **LMPP** | | 0 | 27 | | 14 | | 4 | | 42 | | 0 | | 0 | | 0 | | 0 | | 0 | | 0 | |
| **LMPP-O** | | 0 | 13 | | 4 | | 2 | | 0 | | 54 | | 0 | | 0 | | 0 | | 0 | | 0 | |
| **MEP** | | 0 | 25 | | 0 | | 0 | | 0 | | 0 | | 113 | | 0 | | 0 | | 0 | | 0 | |
| **Mono** | | 0 | 9 | | 1 | | 0 | | 0 | | 0 | | 0 | | 54 | | 0 | | 0 | | 0 | |
| **MPP** | | 0 | 61 | | 0 | | 61 | | 0 | | 0 | | 0 | | 0 | | 20 | | 0 | | 0 | |
| **pDC** | | 0 | 3 | | 5 | | 0 | | 0 | | 0 | | 0 | | 0 | | 0 | | 133 | | 0 | |
| **UNK** | | 1 | 5 | | 22 | | 0 | | 0 | | 0 | | 0 | | 0 | | 0 | | 0 | | 32 | |
| **Enh 0.3 & No Imp** | | | | | | | | | | | | | | | | | | | | | | |
|  | | **CLP** | **CMP** | | **GMP** | | **HSC** | | **LMPP** | | **LMPP-O** | | **MEP** | | **Mono** | | **MPP** | | **pDC** | | **UNK** | |
| **CLP** | | 78 | 0 | | 0 | | 0 | | 0 | | 0 | | 0 | | 0 | | 0 | | 0 | | 0 | |
| **CMP** | | 0 | 465 | | 11 | | 26 | | 0 | | 0 | | 0 | | 0 | | 0 | | 0 | | 0 | |
| **GMP** | | 1 | 44 | | 357 | | 0 | | 0 | | 0 | | 0 | | 0 | | 0 | | 0 | | 0 | |
| **HSC** | | 0 | 63 | | 0 | | 284 | | 0 | | 0 | | 0 | | 0 | | 0 | | 0 | | 0 | |
| **LMPP** | | 0 | 28 | | 12 | | 3 | | 44 | | 0 | | 0 | | 0 | | 0 | | 0 | | 0 | |
| **LMPP-O** | | 0 | 12 | | 4 | | 2 | | 0 | | 55 | | 0 | | 0 | | 0 | | 0 | | 0 | |
| **MEP** | | 0 | 0 | | 0 | | 0 | | 0 | | 0 | | 138 | | 0 | | 0 | | 0 | | 0 | |
| **Mono** | | 0 | 0 | | 4 | | 0 | | 0 | | 0 | | 0 | | 60 | | 0 | | 0 | | 0 | |
| **MPP** | | 0 | 65 | | 0 | | 60 | | 0 | | 0 | | 0 | | 0 | | 17 | | 0 | | 0 | |
| **pDC** | | 0 | 0 | | 5 | | 0 | | 0 | | 0 | | 0 | | 0 | | 0 | | 136 | | 0 | |
| **UNK** | | 1 | 1 | | 17 | | 0 | | 0 | | 0 | | 0 | | 0 | | 0 | | 0 | | 41 | |
| **Enh 0.2 & No Imp** | | | | | | | | | | | | | | | | | | | | | | |
|  | | **CLP** | **CMP** | | **GMP** | | **HSC** | | **LMPP** | | **LMPP-O** | | **MEP** | | **Mono** | | **MPP** | | **pDC** | | **UNK** | |
| **CLP** | | 78 | 0 | | 0 | | 0 | | 0 | | 0 | | 0 | | 0 | | 0 | | 0 | | 0 | |
| **CMP** | | 0 | 467 | | 10 | | 25 | | 0 | | 0 | | 0 | | 0 | | 0 | | 0 | | 0 | |
| **GMP** | | 1 | 50 | | 351 | | 0 | | 0 | | 0 | | 0 | | 0 | | 0 | | 0 | | 0 | |
| **HSC** | | 0 | 68 | | 0 | | 279 | | 0 | | 0 | | 0 | | 0 | | 0 | | 0 | | 0 | |
| **LMPP** | | 0 | 1 | | 2 | | 0 | | 84 | | 0 | | 0 | | 0 | | 0 | | 0 | | 0 | |
| **LMPP-O** | | 0 | 0 | | 1 | | 2 | | 0 | | 70 | | 0 | | 0 | | 0 | | 0 | | 0 | |
| **MEP** | | 0 | 4 | | 0 | | 0 | | 0 | | 0 | | 134 | | 0 | | 0 | | 0 | | 0 | |
| **Mono** | | 0 | 0 | | 5 | | 0 | | 0 | | 0 | | 0 | | 59 | | 0 | | 0 | | 0 | |
| **MPP** | | 0 | 66 | | 0 | | 59 | | 0 | | 0 | | 0 | | 0 | | 17 | | 0 | | 0 | |
| **pDC** | | 0 | 0 | | 9 | | 0 | | 0 | | 0 | | 0 | | 0 | | 0 | | 132 | | 0 | |
| **UNK** | | 0 | 0 | | 5 | | 0 | | 0 | | 0 | | 0 | | 0 | | 0 | | 0 | | 55 | |
| **Enh 0.1 & No Imp** | | | | | | | | | | | | | | | | | | | | | | |
|  | | **CLP** | **CMP** | | **GMP** | | **HSC** | | **LMPP** | | **LMPP-O** | | **MEP** | | **Mono** | | **MPP** | | **pDC** | | **UNK** | |
| **CLP** | | 78 | 0 | | 0 | | 0 | | 0 | | 0 | | 0 | | 0 | | 0 | | 0 | | 0 | |
| **CMP** | | 0 | 502 | | 0 | | 0 | | 0 | | 0 | | 0 | | 0 | | 0 | | 0 | | 0 | |
| **GMP** | | 0 | 0 | | 402 | | 0 | | 0 | | 0 | | 0 | | 0 | | 0 | | 0 | | 0 | |
| **HSC** | | 0 | 0 | | 0 | | 347 | | 0 | | 0 | | 0 | | 0 | | 0 | | 0 | | 0 | |
| **LMPP** | | 0 | 0 | | 0 | | 0 | | 87 | | 0 | | 0 | | 0 | | 0 | | 0 | | 0 | |
| **LMPP-O** | | 0 | 0 | | 0 | | 0 | | 0 | | 73 | | 0 | | 0 | | 0 | | 0 | | 0 | |
| **MEP** | | 0 | 0 | | 0 | | 0 | | 0 | | 0 | | 138 | | 0 | | 0 | | 0 | | 0 | |
| **Mono** | | 0 | 0 | | 0 | | 0 | | 0 | | 0 | | 0 | | 64 | | 0 | | 0 | | 0 | |
| **MPP** | | 0 | 0 | | 0 | | 0 | | 0 | | 0 | | 0 | | 0 | | 142 | | 0 | | 0 | |
| **pDC** | | 0 | 0 | | 0 | | 0 | | 0 | | 0 | | 0 | | 0 | | 0 | | 141 | | 0 | |
| **UNK** | | 0 | 0 | | 0 | | 0 | | 0 | | 0 | | 0 | | 0 | | 0 | | 0 | | 60 | |
| **Enh 0.3 & Imp 0.75** | | | | | | | | | | | | | | | | | | | | | | |
|  | | **CLP** | **CMP** | | **GMP** | | **HSC** | | **LMPP** | | **LMPP-O** | | **MEP** | | **Mono** | | **MPP** | | **pDC** | | **UNK** | |
| **CLP** | | 78 | 0 | | 0 | | 0 | | 0 | | 0 | | 0 | | 0 | | 0 | | 0 | | 0 | |
| **CMP** | | 0 | 466 | | 10 | | 26 | | 0 | | 0 | | 0 | | 0 | | 0 | | 0 | | 0 | |
| **GMP** | | 1 | 44 | | 357 | | 0 | | 0 | | 0 | | 0 | | 0 | | 0 | | 0 | | 0 | |
| **HSC** | | 0 | 64 | | 0 | | 283 | | 0 | | 0 | | 0 | | 0 | | 0 | | 0 | | 0 | |
| **LMPP** | | 0 | 27 | | 12 | | 3 | | 45 | | 0 | | 0 | | 0 | | 0 | | 0 | | 0 | |
| **LMPP-O** | | 0 | 12 | | 4 | | 2 | | 0 | | 55 | | 0 | | 0 | | 0 | | 0 | | 0 | |
| **MEP** | | 0 | 0 | | 0 | | 0 | | 0 | | 0 | | 138 | | 0 | | 0 | | 0 | | 0 | |
| **Mono** | | 0 | 0 | | 4 | | 0 | | 0 | | 0 | | 0 | | 60 | | 0 | | 0 | | 0 | |
| **MPP** | | 0 | 65 | | 0 | | 60 | | 0 | | 0 | | 0 | | 0 | | 17 | | 0 | | 0 | |
| **pDC** | | 0 | 0 | | 6 | | 0 | | 0 | | 0 | | 0 | | 0 | | 0 | | 135 | | 0 | |
| **UNK** | | 1 | 1 | | 17 | | 0 | | 0 | | 0 | | 0 | | 0 | | 0 | | 0 | | 41 | |
| **Enh 0.2 & Imp 0.75** | | | | | | | | | | | | | | | | | | | | | | |
|  | **CLP** | **CMP** | | **GMP** | | **HSC** | | **LMPP** | | **LMPP-O** | | **MEP** | | **Mono** | | **MPP** | | **pDC** | | **UNK** | |  |
| **CLP** | 78 | 0 | | 0 | | 0 | | 0 | | 0 | | 0 | | 0 | | 0 | | 0 | | 0 | |  |
| **CMP** | 0 | 467 | | 10 | | 25 | | 0 | | 0 | | 0 | | 0 | | 0 | | 0 | | 0 | |  |
| **GMP** | 1 | 50 | | 351 | | 0 | | 0 | | 0 | | 0 | | 0 | | 0 | | 0 | | 0 | |  |
| **HSC** | 0 | 68 | | 0 | | 279 | | 0 | | 0 | | 0 | | 0 | | 0 | | 0 | | 0 | |  |
| **LMPP** | 0 | 1 | | 2 | | 0 | | 84 | | 0 | | 0 | | 0 | | 0 | | 0 | | 0 | |  |
| **LMPP-O** | 0 | 0 | | 1 | | 2 | | 0 | | 70 | | 0 | | 0 | | 0 | | 0 | | 0 | |  |
| **MEP** | 0 | 4 | | 0 | | 0 | | 0 | | 0 | | 134 | | 0 | | 0 | | 0 | | 0 | |  |
| **Mono** | 0 | 0 | | 5 | | 0 | | 0 | | 0 | | 0 | | 59 | | 0 | | 0 | | 0 | |  |
| **MPP** | 0 | 66 | | 0 | | 59 | | 0 | | 0 | | 0 | | 0 | | 17 | | 0 | | 0 | |  |
| **pDC** | 0 | 0 | | 9 | | 0 | | 0 | | 0 | | 0 | | 0 | | 0 | | 132 | | 0 | |  |
| **UNK** | 0 | 0 | | 5 | | 0 | | 0 | | 0 | | 0 | | 0 | | 0 | | 0 | | 55 | |  |
| **Enh 0.1 & Imp 0.75** | | | | | | | | | | | | | | | | | | | | | |  |
|  | **CLP** | **CMP** | | **GMP** | | **HSC** | | **LMPP** | | **LMPP-O** | | **MEP** | | **Mono** | | **MPP** | | **pDC** | | **UNK** | |  |
| **CLP** | 78 | 0 | | 0 | | 0 | | 0 | | 0 | | 0 | | 0 | | 0 | | 0 | | 0 | |  |
| **CMP** | 0 | 502 | | 0 | | 0 | | 0 | | 0 | | 0 | | 0 | | 0 | | 0 | | 0 | |  |
| **GMP** | 0 | 0 | | 402 | | 0 | | 0 | | 0 | | 0 | | 0 | | 0 | | 0 | | 0 | |  |
| **HSC** | 0 | 0 | | 0 | | 347 | | 0 | | 0 | | 0 | | 0 | | 0 | | 0 | | 0 | |  |
| **LMPP** | 0 | 0 | | 0 | | 0 | | 87 | | 0 | | 0 | | 0 | | 0 | | 0 | | 0 | |  |
| **LMPP-O** | 0 | 0 | | 0 | | 0 | | 0 | | 73 | | 0 | | 0 | | 0 | | 0 | | 0 | |  |
| **MEP** | 0 | 0 | | 0 | | 0 | | 0 | | 0 | | 138 | | 0 | | 0 | | 0 | | 0 | |  |
| **Mono** | 0 | 0 | | 0 | | 0 | | 0 | | 0 | | 0 | | 64 | | 0 | | 0 | | 0 | |  |
| **MPP** | 0 | 0 | | 0 | | 0 | | 0 | | 0 | | 0 | | 0 | | 142 | | 0 | | 0 | |  |
| **pDC** | 0 | 0 | | 0 | | 0 | | 0 | | 0 | | 0 | | 0 | | 0 | | 141 | | 0 | |  |
| **UNK** | 0 | 0 | | 0 | | 0 | | 0 | | 0 | | 0 | | 0 | | 0 | | 0 | | 60 | |  |
| **Enh 0.3 & Imp 0.5** | | | | | | | | | | | | | | | | | | | | | |  |
|  | **CLP** | **CMP** | | **GMP** | | **HSC** | | **LMPP** | | **LMPP-O** | | **MEP** | | **Mono** | | **MPP** | | **pDC** | | **UNK** | |  |
| **CLP** | 78 | 0 | | 0 | | 0 | | 0 | | 0 | | 0 | | 0 | | 0 | | 0 | | 0 | |  |
| **CMP** | 0 | 467 | | 10 | | 25 | | 0 | | 0 | | 0 | | 0 | | 0 | | 0 | | 0 | |  |
| **GMP** | 1 | 46 | | 355 | | 0 | | 0 | | 0 | | 0 | | 0 | | 0 | | 0 | | 0 | |  |
| **HSC** | 0 | 65 | | 0 | | 282 | | 0 | | 0 | | 0 | | 0 | | 0 | | 0 | | 0 | |  |
| **LMPP** | 0 | 29 | | 13 | | 3 | | 42 | | 0 | | 0 | | 0 | | 0 | | 0 | | 0 | |  |
| **LMPP-O** | 0 | 12 | | 4 | | 2 | | 0 | | 55 | | 0 | | 0 | | 0 | | 0 | | 0 | |  |
| **MEP** | 0 | 1 | | 0 | | 0 | | 0 | | 0 | | 137 | | 0 | | 0 | | 0 | | 0 | |  |
| **Mono** | 0 | 0 | | 5 | | 0 | | 0 | | 0 | | 0 | | 59 | | 0 | | 0 | | 0 | |  |
| **MPP** | 0 | 65 | | 0 | | 60 | | 0 | | 0 | | 0 | | 0 | | 17 | | 0 | | 0 | |  |
| **pDC** | 0 | 0 | | 9 | | 0 | | 0 | | 0 | | 0 | | 0 | | 0 | | 132 | | 0 | |  |
| **UNK** | 0 | 0 | | 12 | | 0 | | 0 | | 0 | | 0 | | 0 | | 0 | | 0 | | 48 | |  |
| **Enh 0.2 & Imp 0.5** | | | | | | | | | | | | | | | | | | | | | |  |
|  | **CLP** | **CMP** | | **GMP** | | **HSC** | | **LMPP** | | **LMPP-O** | | **MEP** | | **Mono** | | **MPP** | | **pDC** | | **UNK** | |  |
| **CLP** | 78 | 0 | | 0 | | 0 | | 0 | | 0 | | 0 | | 0 | | 0 | | 0 | | 0 | |  |
| **CMP** | 0 | 467 | | 10 | | 25 | | 0 | | 0 | | 0 | | 0 | | 0 | | 0 | | 0 | |  |
| **GMP** | 0 | 51 | | 351 | | 0 | | 0 | | 0 | | 0 | | 0 | | 0 | | 0 | | 0 | |  |
| **HSC** | 0 | 68 | | 0 | | 279 | | 0 | | 0 | | 0 | | 0 | | 0 | | 0 | | 0 | |  |
| **LMPP** | 0 | 0 | | 2 | | 0 | | 85 | | 0 | | 0 | | 0 | | 0 | | 0 | | 0 | |  |
| **LMPP-O** | 0 | 0 | | 1 | | 2 | | 0 | | 70 | | 0 | | 0 | | 0 | | 0 | | 0 | |  |
| **MEP** | 0 | 4 | | 0 | | 0 | | 0 | | 0 | | 134 | | 0 | | 0 | | 0 | | 0 | |  |
| **Mono** | 0 | 0 | | 5 | | 0 | | 0 | | 0 | | 0 | | 59 | | 0 | | 0 | | 0 | |  |
| **MPP** | 0 | 66 | | 0 | | 59 | | 0 | | 0 | | 0 | | 0 | | 17 | | 0 | | 0 | |  |
| **pDC** | 0 | 0 | | 7 | | 0 | | 0 | | 0 | | 0 | | 0 | | 0 | | 134 | | 0 | |  |
| **UNK** | 0 | 0 | | 5 | | 0 | | 0 | | 0 | | 0 | | 0 | | 0 | | 0 | | 55 | |  |
| **Enh 0.1 & Imp 0.5** | | | | | | | | | | | | | | | | | | | | | |  |
|  | **CLP** | **CMP** | | **GMP** | | **HSC** | | **LMPP** | | **LMPP-O** | | **MEP** | | **Mono** | | **MPP** | | **pDC** | | **UNK** | |  |
| **CLP** | 78 | 0 | | 0 | | 0 | | 0 | | 0 | | 0 | | 0 | | 0 | | 0 | | 0 | |  |
| **CMP** | 0 | 502 | | 0 | | 0 | | 0 | | 0 | | 0 | | 0 | | 0 | | 0 | | 0 | |  |
| **GMP** | 0 | 0 | | 402 | | 0 | | 0 | | 0 | | 0 | | 0 | | 0 | | 0 | | 0 | |  |
| **HSC** | 0 | 0 | | 0 | | 347 | | 0 | | 0 | | 0 | | 0 | | 0 | | 0 | | 0 | |  |
| **LMPP** | 0 | 0 | | 0 | | 0 | | 87 | | 0 | | 0 | | 0 | | 0 | | 0 | | 0 | |  |
| **LMPP-O** | 0 | 0 | | 0 | | 0 | | 0 | | 73 | | 0 | | 0 | | 0 | | 0 | | 0 | |  |
| **MEP** | 0 | 0 | | 0 | | 0 | | 0 | | 0 | | 138 | | 0 | | 0 | | 0 | | 0 | |  |
| **Mono** | 0 | 0 | | 0 | | 0 | | 0 | | 0 | | 0 | | 64 | | 0 | | 0 | | 0 | |  |
| **MPP** | 0 | 0 | | 0 | | 0 | | 0 | | 0 | | 0 | | 0 | | 142 | | 0 | | 0 | |  |
| **pDC** | 0 | 0 | | 0 | | 0 | | 0 | | 0 | | 0 | | 0 | | 0 | | 141 | | 0 | |  |
| **UNK** | 0 | 0 | | 0 | | 0 | | 0 | | 0 | | 0 | | 0 | | 0 | | 0 | | 60 | |  |
| **Enh 0.3 & Imp 0.25** | | | | | | | | | | | | | | | | | | | | | |  |
|  | **CLP** | **CMP** | | **GMP** | | **HSC** | | **LMPP** | | **LMPP-O** | | **MEP** | | **Mono** | | **MPP** | | **pDC** | | **UNK** | |  |
| **CLP** | 78 | 0 | | 0 | | 0 | | 0 | | 0 | | 0 | | 0 | | 0 | | 0 | | 0 | |  |
| **CMP** | 0 | 468 | | 10 | | 24 | | 0 | | 0 | | 0 | | 0 | | 0 | | 0 | | 0 | |  |
| **GMP** | 0 | 47 | | 355 | | 0 | | 0 | | 0 | | 0 | | 0 | | 0 | | 0 | | 0 | |  |
| **HSC** | 0 | 65 | | 0 | | 282 | | 0 | | 0 | | 0 | | 0 | | 0 | | 0 | | 0 | |  |
| **LMPP** | 0 | 30 | | 13 | | 3 | | 41 | | 0 | | 0 | | 0 | | 0 | | 0 | | 0 | |  |
| **LMPP-O** | 0 | 5 | | 3 | | 2 | | 0 | | 63 | | 0 | | 0 | | 0 | | 0 | | 0 | |  |
| **MEP** | 0 | 3 | | 0 | | 0 | | 0 | | 0 | | 135 | | 0 | | 0 | | 0 | | 0 | |  |
| **Mono** | 0 | 0 | | 5 | | 0 | | 0 | | 0 | | 0 | | 59 | | 0 | | 0 | | 0 | |  |
| **MPP** | 0 | 65 | | 0 | | 60 | | 0 | | 0 | | 0 | | 0 | | 17 | | 0 | | 0 | |  |
| **pDC** | 0 | 0 | | 7 | | 0 | | 0 | | 0 | | 0 | | 0 | | 0 | | 134 | | 0 | |  |
| **UNK** | 0 | 0 | | 6 | | 0 | | 0 | | 0 | | 0 | | 0 | | 0 | | 0 | | 54 | |  |
| **Enh 0.2 & Imp 0.25** | | | | | | | | | | | | | | | | | | | | | |  |
|  | **CLP** | **CMP** | | **GMP** | | **HSC** | | **LMPP** | | **LMPP-O** | | **MEP** | | **Mono** | | **MPP** | | **pDC** | | **UNK** | |  |
| **CLP** | 78 | 0 | | 0 | | 0 | | 0 | | 0 | | 0 | | 0 | | 0 | | 0 | | 0 | |  |
| **CMP** | 0 | 469 | | 10 | | 23 | | 0 | | 0 | | 0 | | 0 | | 0 | | 0 | | 0 | |  |
| **GMP** | 0 | 51 | | 351 | | 0 | | 0 | | 0 | | 0 | | 0 | | 0 | | 0 | | 0 | |  |
| **HSC** | 0 | 69 | | 0 | | 278 | | 0 | | 0 | | 0 | | 0 | | 0 | | 0 | | 0 | |  |
| **LMPP** | 0 | 0 | | 2 | | 0 | | 85 | | 0 | | 0 | | 0 | | 0 | | 0 | | 0 | |  |
| **LMPP-O** | 0 | 0 | | 0 | | 0 | | 0 | | 73 | | 0 | | 0 | | 0 | | 0 | | 0 | |  |
| **MEP** | 0 | 4 | | 0 | | 0 | | 0 | | 0 | | 134 | | 0 | | 0 | | 0 | | 0 | |  |
| **Mono** | 0 | 0 | | 4 | | 0 | | 0 | | 0 | | 0 | | 60 | | 0 | | 0 | | 0 | |  |
| **MPP** | 0 | 66 | | 0 | | 59 | | 0 | | 0 | | 0 | | 0 | | 17 | | 0 | | 0 | |  |
| **pDC** | 0 | 0 | | 5 | | 0 | | 0 | | 0 | | 0 | | 0 | | 0 | | 136 | | 0 | |  |
| **UNK** | 0 | 0 | | 4 | | 0 | | 0 | | 0 | | 0 | | 0 | | 0 | | 0 | | 56 | |  |
| **Enh 0.1 & Imp 0.25** | | | | | | | | | | | | | | | | | | | | | |  |
|  | **CLP** | **CMP** | | **GMP** | | **HSC** | | **LMPP** | | **LMPP-O** | | **MEP** | | **Mono** | | **MPP** | | **pDC** | | **UNK** | |  |
| **CLP** | 78 | 0 | | 0 | | 0 | | 0 | | 0 | | 0 | | 0 | | 0 | | 0 | | 0 | |  |
| **CMP** | 0 | 502 | | 0 | | 0 | | 0 | | 0 | | 0 | | 0 | | 0 | | 0 | | 0 | |  |
| **GMP** | 0 | 0 | | 402 | | 0 | | 0 | | 0 | | 0 | | 0 | | 0 | | 0 | | 0 | |  |
| **HSC** | 0 | 0 | | 0 | | 347 | | 0 | | 0 | | 0 | | 0 | | 0 | | 0 | | 0 | |  |
| **LMPP** | 0 | 0 | | 0 | | 0 | | 87 | | 0 | | 0 | | 0 | | 0 | | 0 | | 0 | |  |
| **LMPP-O** | 0 | 0 | | 0 | | 0 | | 0 | | 73 | | 0 | | 0 | | 0 | | 0 | | 0 | |  |
| **MEP** | 0 | 0 | | 0 | | 0 | | 0 | | 0 | | 138 | | 0 | | 0 | | 0 | | 0 | |  |
| **Mono** | 0 | 0 | | 0 | | 0 | | 0 | | 0 | | 0 | | 64 | | 0 | | 0 | | 0 | |  |
| **MPP** | 0 | 0 | | 0 | | 0 | | 0 | | 0 | | 0 | | 0 | | 142 | | 0 | | 0 | |  |
| **pDC** | 0 | 0 | | 0 | | 0 | | 0 | | 0 | | 0 | | 0 | | 0 | | 141 | | 0 | |  |
| **UNK** | 0 | 0 | | 0 | | 0 | | 0 | | 0 | | 0 | | 0 | | 0 | | 0 | | 60 | |  |

*Note*: In each table, the row represents the true label of cells and column represents the predicted label of cells
