# Supplementary material for: Enhancement and Imputation of Peak Signal Enables Accurate Cell-Type Classification in scATAC-seq
Source: Front Genet. 2021 Apr 6;12:658352. doi: 10.3389/fgene.2021.658352 (PMC8056015; doi:10.3389/fgene.2021.658352)
Supplement: Supplementary Table 5 — F1 scores of intra-dataset experiment using 10× PBMCs v1 Seurat Labeled dataset with different enhancement and imputation cutoffs. [file Table_5.DOCX]

**Supplementary Table 5 F1 scores of intra-dataset experiment using 10x PBMCs v1 Seurat Labelled dataset with different enhancement and imputation cutoffs**

| **F1 score** | **B** | **CD14+ Mono** | **CD8+ T** | **DC** | **FCGR3A+ Mono** | **Memory CD4+** | **Naive CD4+ T** |
| --- | --- | --- | --- | --- | --- | --- | --- |
| No Enhancement & No Imputation | 0.9886914 | 0.95446266 | 0 | 0.0869565 | 0 | 0.90497738 | 0.89202541 |
| Enh 0.3 & No Imp | 0 | 0.61231361 | 0 | 0 | 0 | 0 | 0.04722222 |
| Enh 0.3 & Imp 0.75 | 0.9934853 | 0.96255507 | 0 | 0.9767442 | 0.05940594 | 0.90519187 | 0.89280677 |
| Enh 0.3 & Imp 0.5 | 0.9934853 | 0.96788483 | 0 | 0.9767442 | 0.31034483 | 0.90519187 | 0.89280677 |
| Enh 0.3 & Imp 0.25 | 0.9918434 | 0.99055535 | 0 | 0.9767442 | 0.91111111 | 0.9039548 | 0.89139633 |
| Enh 0.2 & No Imp | 0 | 0.6119403 | 0 | 0 | 0 | 0 | 0.04702628 |
| Enh 0.2 & Imp 0.75 | 0.9918434 | 0.99130435 | 0.4719101 | 0.9767442 | 0.93478261 | 0.98504274 | 0.95089286 |
| Enh 0.2 & Imp 0.5 | 0.9885434 | 0.99091597 | 0.9291339 | 0.9767442 | 0.95187166 | 0.99148936 | 0.98159509 |
| Enh 0.2 & Imp 0.25 | 0.99019608 | 0.992794843 | 1 | 1 | 0.962962963 | 0.993630573 | 0.989180835 |
| Enh 0.1 & No Imp | 0 | 0.61249096 | 0 | 0 | 0 | 0 | 0.04155125 |
| Enh 0.1 & Imp 0.75 | 1 | 1 | 1 | 1 | 1 | 1 | 1 |
| Enh 0.1 & Imp 0.5 | 1 | 1 | 1 | 1 | 1 | 1 | 1 |
| Enh 0.1 & Imp 0.25 | 1 | 1 | 1 | 1 | 1 | 1 | 1 |
