# Supplementary material for: Enhancement and Imputation of Peak Signal Enables Accurate Cell-Type Classification in scATAC-seq
Source: Front Genet. 2021 Apr 6;12:658352. doi: 10.3389/fgene.2021.658352 (PMC8056015; doi:10.3389/fgene.2021.658352)
Supplement: Supplementary Table 6 — The confusion matrix across different enhancement and imputation cutoffs for 10× PBMCs v1 Seurat Labeled dataset. [file Table_6.DOCX]

**Supplementary Table 6 The confusion matrix across different enhancement and imputation cutoffs for 10x PBMCs v1 Seurat Labelled dataset**

| **No Enh & No Imp** | | | | | | | |
| --- | --- | --- | --- | --- | --- | --- | --- |
|  | **B** | **CD14+ Mono** | **CD8+ T** | **DC** | **FCGR3A+ Mono** | **Memory CD4+** | **Naive CD4+ T** |
| **B** | 306 | 1 |  | 0 |  | 2 | 0 |
| **CD14+ Mono** | 1 | 1310 |  | 0 |  | 0 | 3 |
| **CD8+ T** | 0 | 0 |  | 0 |  | 0 | 68 |
| **DC** | 1 | 19 |  | 1 |  | 0 | 1 |
| **FCGR3A+ Mono** | 1 | 96 |  | 0 |  | 1 | 0 |
| **Memory CD4+** | 1 | 2 |  | 0 |  | 400 | 71 |
| **Naive CD4+ T** | 0 | 3 |  | 0 |  | 7 | 632 |
| **Enh 0.3 & No Imp** | | | | | | | |
|  | **B** | **CD14+ Mono** | **CD8+ T** | **DC** | **FCGR3A+ Mono** | **Memory CD4+** | **Naive CD4+ T** |
| **B** | 0 | 298 | 0 | 0 | 0 | 0 | 11 |
| **CD14+ Mono** | 0 | 1273 | 0 | 0 | 0 | 3 | 38 |
| **CD8+ T** | 0 | 67 | 0 | 0 | 0 | 1 | 0 |
| **DC** | 0 | 21 | 0 | 0 | 0 | 0 | 1 |
| **FCGR3A+ Mono** | 0 | 94 | 0 | 0 | 0 | 0 | 4 |
| **Memory CD4+** | 0 | 467 | 0 | 0 | 0 | 0 | 7 |
| **Naive CD4+ T** | 0 | 624 | 0 | 0 | 0 | 1 | 17 |
| **Enh 0.2 & No Imp** | | | | | | | |
|  | **B** | **CD14+ Mono** | **CD8+ T** | **DC** | **FCGR3A+ Mono** | **Memory CD4+** | **Naive CD4+ T** |
| **B** | 0 | 297 | 0 | 0 | 0 | 0 | 12 |
| **CD14+ Mono** | 0 | 1271 | 0 | 0 | 0 | 4 | 39 |
| **CD8+ T** | 0 | 66 | 0 | 0 | 0 | 1 | 1 |
| **DC** | 0 | 21 | 0 | 0 | 0 | 0 | 1 |
| **FCGR3A+ Mono** | 0 | 94 | 0 | 0 | 0 | 0 | 4 |
| **Memory CD4+** | 0 | 467 | 0 | 0 | 0 | 0 | 7 |
| **Naive CD4+ T** | 0 | 624 | 0 | 0 | 0 | 1 | 17 |
| **Enh 0.1 & No Imp** | | | | | | | |
|  | **B** | **CD14+ Mono** | **CD8+ T** | **DC** | **FCGR3A+ Mono** | **Memory CD4+** | **Naive CD4+ T** |
| **B** | 0 | 290 | 0 | 0 | 0 | 1 | 18 |
| **CD14+ Mono** | 1 | 1270 | 0 | 0 | 0 | 10 | 33 |
| **CD8+ T** | 0 | 66 | 0 | 0 | 0 | 0 | 2 |
| **DC** | 0 | 21 | 0 | 0 | 0 | 0 | 1 |
| **FCGR3A+ Mono** | 0 | 96 | 0 | 0 | 0 | 0 | 2 |
| **Memory CD4+** | 0 | 465 | 0 | 0 | 0 | 0 | 9 |
| **Naive CD4+ T** | 0 | 625 | 0 | 0 | 0 | 2 | 15 |
| **Enh 0.3 & Imp 0.75** | | | | | | | |
|  | **B** | **CD14+ Mono** | **CD8+ T** | **DC** | **FCGR3A+ Mono** | **Memory CD4+** | **Naive CD4+ T** |
| **B** | 305 | 2 | 0 | 0 | 0 | 2 | 0 |
| **CD14+ Mono** | 0 | 1311 | 0 | 0 | 0 | 0 | 3 |
| **CD8+ T** | 0 | 0 | 0 | 0 | 0 | 0 | 68 |
| **DC** | 0 | 0 | 0 | 21 | 0 | 0 | 1 |
| **FCGR3A+ Mono** | 0 | 93 | 0 | 0 | 3 | 2 | 0 |
| **Memory CD4+** | 0 | 2 | 0 | 0 | 0 | 401 | 71 |
| **Naive CD4+ T** | 0 | 2 | 0 | 0 | 0 | 7 | 633 |
| **Enh 0.2 & Imp 0.75** | | | | | | | |
|  | **B** | **CD14+ Mono** | **CD8+ T** | **DC** | **FCGR3A+ Mono** | **Memory CD4+** | **Naive CD4+ T** |
| **B** | 304 | 4 | 0 | 0 | 0 | 0 | 1 |
| **CD14+ Mono** | 0 | 1311 | 0 | 0 | 0 | 0 | 3 |
| **CD8+ T** | 0 | 0 | 21 | 0 | 0 | 0 | 47 |
| **DC** | 0 | 0 | 0 | 21 | 0 | 0 | 1 |
| **FCGR3A+ Mono** | 0 | 12 | 0 | 0 | 86 | 0 | 0 |
| **Memory CD4+** | 0 | 2 | 0 | 0 | 0 | 461 | 11 |
| **Naive CD4+ T** | 0 | 2 | 0 | 0 | 0 | 1 | 639 |
| **Enh 0.1 & Imp 0.75** | | | | | | | |
|  | **B** | **CD14+ Mono** | **CD8+ T** | **DC** | **FCGR3A+ Mono** | **Memory CD4+** | **Naive CD4+ T** |
| **B** | 309 | 0 | 0 | 0 | 0 | 0 | 0 |
| **CD14+ Mono** | 0 | 1314 | 0 | 0 | 0 | 0 | 0 |
| **CD8+ T** | 0 | 0 | 68 | 0 | 0 | 0 | 0 |
| **DC** | 0 | 0 | 0 | 22 | 0 | 0 | 0 |
| **FCGR3A+ Mono** | 0 | 0 | 0 | 0 | 98 | 0 | 0 |
| **Memory CD4+** | 0 | 0 | 0 | 0 | 0 | 474 | 0 |
| **Naive CD4+ T** | 0 | 0 | 0 | 0 | 0 | 0 | 642 |
| **Enh 0.3 & Imp 0.5** | | | | | | | |
|  | **B** | **CD14+ Mono** | **CD8+ T** | **DC** | **FCGR3A+ Mono** | **Memory CD4+** | **Naive CD4+ T** |
| **B** | 305 | 2 | 0 | 0 | 0 | 2 | 0 |
| **CD14+ Mono** | 0 | 1311 | 0 | 0 | 0 | 0 | 3 |
| **CD8+ T** | 0 | 0 | 0 | 0 | 0 | 0 | 68 |
| **DC** | 0 | 0 | 0 | 21 | 0 | 0 | 1 |
| **FCGR3A+ Mono** | 0 | 78 | 0 | 0 | 18 | 2 | 0 |
| **Memory CD4+** | 0 | 2 | 0 | 0 | 0 | 401 | 71 |
| **Naive CD4+ T** | 0 | 2 | 0 | 0 | 0 | 7 | 633 |
| **Enh 0.2 & Imp 0.5** | | | | | | | |
|  | **B** | **CD14+ Mono** | **CD8+ T** | **DC** | **FCGR3A+ Mono** | **Memory CD4+** | **Naive CD4+ T** |
| **B** | 302 | 6 | 0 | 0 | 0 | 0 | 1 |
| **CD14+ Mono** | 0 | 1309 | 0 | 0 | 0 | 0 | 5 |
| **CD8+ T** | 0 | 0 | 59 | 0 | 0 | 0 | 9 |
| **DC** | 0 | 0 | 0 | 21 | 0 | 0 | 1 |
| **FCGR3A+ Mono** | 0 | 9 | 0 | 0 | 89 | 0 | 0 |
| **Memory CD4+** | 0 | 2 | 0 | 0 | 0 | 466 | 6 |
| **Naive CD4+ T** | 0 | 2 | 0 | 0 | 0 | 0 | 640 |
| **Enh 0.1 & Imp 0.5** | | | | | | | |
|  | **B** | **CD14+ Mono** | **CD8+ T** | **DC** | **FCGR3A+ Mono** | **Memory CD4+** | **Naive CD4+ T** |
| **B** | 309 | 0 | 0 | 0 | 0 | 0 | 0 |
| **CD14+ Mono** | 0 | 1314 | 0 | 0 | 0 | 0 | 0 |
| **CD8+ T** | 0 | 0 | 68 | 0 | 0 | 0 | 0 |
| **DC** | 0 | 0 | 0 | 22 | 0 | 0 | 0 |
| **FCGR3A+ Mono** | 0 | 0 | 0 | 0 | 98 | 0 | 0 |
| **Memory CD4+** | 0 | 0 | 0 | 0 | 0 | 474 | 0 |
| **Naive CD4+ T** | 0 | 0 | 0 | 0 | 0 | 0 | 642 |
| **Enh 0.3 & Imp 0.25** | | | | | | | |
|  | **B** | **CD14+ Mono** | **CD8+ T** | **DC** | **FCGR3A+ Mono** | **Memory CD4+** | **Naive CD4+ T** |
| **B** | 304 | 3 | 0 | 0 | 0 | 2 | 0 |
| **CD14+ Mono** | 0 | 1311 | 0 | 0 | 0 | 0 | 3 |
| **CD8+ T** | 0 | 0 | 0 | 0 | 0 | 0 | 68 |
| **DC** | 0 | 0 | 0 | 21 | 0 | 0 | 1 |
| **FCGR3A+ Mono** | 0 | 14 | 0 | 0 | 82 | 2 | 0 |
| **Memory CD4+** | 0 | 2 | 0 | 0 | 0 | 400 | 72 |
| **Naive CD4+ T** | 0 | 3 | 0 | 0 | 0 | 7 | 632 |
| **Enh 0.2 & Imp 0.25** | | | | | | | |
|  | **B** | **CD14+ Mono** | **CD8+ T** | **DC** | **FCGR3A+ Mono** | **Memory CD4+** | **Naive CD4+ T** |
| **B** | 303 | 4 | 0 | 0 | 0 | 0 | 2 |
| **CD14+ Mono** | 0 | 1309 | 0 | 0 | 0 | 0 | 5 |
| **CD8+ T** | 0 | 0 | 68 | 0 | 0 | 0 | 0 |
| **DC** | 0 | 0 | 0 | 22 | 0 | 0 | 0 |
| **FCGR3A+ Mono** | 0 | 7 | 0 | 0 | 91 | 0 | 0 |
| **Memory CD4+** | 0 | 1 | 0 | 0 | 0 | 468 | 5 |
| **Naive CD4+ T** | 0 | 2 | 0 | 0 | 0 | 0 | 640 |
| **Enh 0.1 & Imp 0.25** | | | | | | | |
|  | **B** | **CD14+ Mono** | **CD8+ T** | **DC** | **FCGR3A+ Mono** | **Memory CD4+** | **Naive CD4+ T** |
| **B** | 309 | 0 | 0 | 0 | 0 | 0 | 0 |
| **CD14+ Mono** | 0 | 1314 | 0 | 0 | 0 | 0 | 0 |
| **CD8+ T** | 0 | 0 | 68 | 0 | 0 | 0 | 0 |
| **DC** | 0 | 0 | 0 | 22 | 0 | 0 | 0 |
| **FCGR3A+ Mono** | 0 | 0 | 0 | 0 | 98 | 0 | 0 |
| **Memory CD4+** | 0 | 0 | 0 | 0 | 0 | 474 | 0 |
| **Naive CD4+ T** | 0 | 0 | 0 | 0 | 0 | 0 | 642 |

*Note*: In each table, the row represents the true label of cells and column represents the predicted label of cells
