# Supplementary material for: Enhancement and Imputation of Peak Signal Enables Accurate Cell-Type Classification in scATAC-seq
Source: Front Genet. 2021 Apr 6;12:658352. doi: 10.3389/fgene.2021.658352 (PMC8056015; doi:10.3389/fgene.2021.658352)
Supplement: Supplementary Table 7 — F1 scores of intra-dataset experiment using 10× PBMCs Next Gem Seurat Labeled dataset with different enhancement and imputation cutoffs. [file Table_7.DOCX]

**Supplementary Table 7 F1 scores of intra-dataset experiment using 10x PBMCs Next Gem Seurat Labelled dataset with different enhancement and imputation cutoffs**

| **F1 score** | **B** | **CD14+ Mono** | **CD8+ T** | **DC** | **FCGR3A+ Mono** | **Memory CD4+** | **Naive CD4+ T** |
| --- | --- | --- | --- | --- | --- | --- | --- |
| No Enhancement & No Imputation | 0.9954649 | 0.96742471 | 0.1626016 | 0.8571429 | 0.49425287 | 0.91036633 | 0.79307958 |
| Enh 0.3 & No Imp | 0.0044444 | 0.59427904 | 0 | 0 | 0 | 0.02349486 | 0.02639296 |
| Enh 0.3 & Imp 0.75 | 0.9977324 | 0.99057789 | 0.9208633 | 0.9677419 | 0.9218107 | 0.98342541 | 0.96342412 |
| Enh 0.3 & Imp 0.5 | 0.9977324 | 0.99432535 | 0.9749431 | 0.9677419 | 0.97254902 | 0.98970705 | 0.98658248 |
| Enh 0.3 & Imp 0.25 | 0.9988675 | 0.99558081 | 0.9841987 | 0.9677419 | 0.98449612 | 0.99129058 | 0.98970705 |
| Enh 0.2 & No Imp | 0.0044543 | 0.59377432 | 0 | 0 | 0 | 0.02877698 | 0.0239521 |
| Enh 0.2 & Imp 0.75 | 0.9988675 | 1 | 1 | 1 | 1 | 0.99921569 | 1 |
| Enh 0.2 & Imp 0.5 | 1 | 1 | 1 | 1 | 1 | 1 | 1 |
| Enh 0.2 & Imp 0.25 | 1 | 1 | 1 | 1 | 1 | 1 | 1 |
| Enh 0.1 & No Imp | 0 | 0.59945925 | 0 | 0 | 0 | 0.02080238 | 0.0152439 |
| Enh 0.1 & Imp 0.75 | 1 | 1 | 1 | 1 | 1 | 1 | 1 |
| Enh 0.1 & Imp 0.5 | 1 | 1 | 1 | 1 | 1 | 1 | 1 |
| Enh 0.1 & Imp 0.25 | 1 | 1 | 1 | 1 | 1 | 1 | 1 |
