# Supplementary material for: Enhancement and Imputation of Peak Signal Enables Accurate Cell-Type Classification in scATAC-seq
Source: Front Genet. 2021 Apr 6;12:658352. doi: 10.3389/fgene.2021.658352 (PMC8056015; doi:10.3389/fgene.2021.658352)
Supplement: Supplementary Table 8 — The confusion matrix across different enhancement and imputation cutoffs for 10× PBMCs Next Gem Seurat Labeled dataset. [file Table_8.DOCX]

**Supplementary Table 8 The confusion matrix across different enhancement and imputation cutoffs for 10x PBMCs Next Gem Seurat Labelled dataset**

| **No Enh & No Imp** | | | | | | | |
| --- | --- | --- | --- | --- | --- | --- | --- |
|  | **B** | **CD14+ Mono** | **CD8+ T** | **DC** | **FCGR3A+ Mono** | **Memory CD4+** | **Naive CD4+ T** |
| **B** | 439 | 1 | 0 | 0 | 0 | 2 | 0 |
| **CD14+ Mono** | 0 | 1574 | 0 | 0 | 0 | 3 | 0 |
| **CD8+ T** | 0 | 1 | 20 | 0 | 0 | 7 | 197 |
| **DC** | 0 | 7 | 0 | 24 | 0 | 1 | 0 |
| **FCGR3A+ Mono** | 0 | 88 | 0 | 0 | 43 | 0 | 0 |
| **Memory CD4+** | 0 | 4 | 0 | 0 | 0 | 584 | 49 |
| **Naive CD4+ T** | 1 | 2 | 1 | 0 | 0 | 49 | 573 |
| **Enh 0.3 & No Imp** | | | | | | | |
|  | **B** | **CD14+ Mono** | **CD8+ T** | **DC** | **FCGR3A+ Mono** | **Memory CD4+** | **Naive CD4+ T** |
| **B** | 1 | 431 | 0 | 0 | 0 | 4 | 6 |
| **CD14+ Mono** | 1 | 1527 | 0 | 0 | 0 | 20 | 29 |
| **CD8+ T** | 2 | 218 | 0 | 0 | 0 | 2 | 3 |
| **DC** | 0 | 31 | 0 | 0 | 0 | 0 | 1 |
| **FCGR3A+ Mono** | 0 | 125 | 0 | 0 | 0 | 4 | 2 |
| **Memory CD4+** | 3 | 620 | 0 | 0 | 0 | 8 | 6 |
| **Naive CD4+ T** | 1 | 610 | 0 | 0 | 0 | 6 | 9 |
| **Enh 0.2 & No Imp** | | | | | | | |
|  | **B** | **CD14+ Mono** | **CD8+ T** | **DC** | **FCGR3A+ Mono** | **Memory CD4+** | **Naive CD4+ T** |
| **B** | 1 | 432 | 0 | 0 | 0 | 4 | 5 |
| **CD14+ Mono** | 2 | 1526 | 0 | 0 | 0 | 31 | 18 |
| **CD8+ T** | 1 | 219 | 0 | 0 | 0 | 1 | 4 |
| **DC** | 0 | 31 | 0 | 0 | 0 | 0 | 1 |
| **FCGR3A+ Mono** | 1 | 127 | 0 | 0 | 0 | 2 | 1 |
| **Memory CD4+** | 2 | 620 | 0 | 0 | 0 | 10 | 5 |
| **Naive CD4+ T** | 0 | 608 | 0 | 0 | 0 | 10 | 8 |
| **Enh 0.1 & No Imp** | | | | | | | |
|  | **B** | **CD14+ Mono** | **CD8+ T** | **DC** | **FCGR3A+ Mono** | **Memory CD4+** | **Naive CD4+ T** |
| **B** | 0 | 437 | 0 | 0 | 0 | 2 | 3 |
| **CD14+ Mono** | 1 | 1552 | 0 | 0 | 0 | 10 | 14 |
| **CD8+ T** | 1 | 217 | 0 | 0 | 0 | 2 | 5 |
| **DC** | 0 | 32 | 0 | 0 | 0 | 0 | 0 |
| **FCGR3A+ Mono** | 0 | 127 | 0 | 0 | 0 | 4 | 0 |
| **Memory CD4+** | 1 | 626 | 0 | 0 | 0 | 7 | 3 |
| **Naive CD4+ T** | 0 | 610 | 0 | 0 | 0 | 11 | 5 |
| **Enh 0.3 & Imp 0.75** | | | | | | | |
|  | **B** | **CD14+ Mono** | **CD8+ T** | **DC** | **FCGR3A+ Mono** | **Memory CD4+** | **Naive CD4+ T** |
| **B** | 440 | 1 | 0 | 0 | 0 | 1 | 0 |
| **CD14+ Mono** | 0 | 1577 | 0 | 0 | 0 | 0 | 0 |
| **CD8+ T** | 0 | 1 | 192 | 0 | 0 | 0 | 32 |
| **DC** | 0 | 2 | 0 | 30 | 0 | 0 | 0 |
| **FCGR3A+ Mono** | 0 | 19 | 0 | 0 | 112 | 0 | 0 |
| **Memory CD4+** | 0 | 6 | 0 | 0 | 0 | 623 | 8 |
| **Naive CD4+ T** | 0 | 1 | 0 | 0 | 0 | 6 | 619 |
| **Enh 0.2 & Imp 0.75** | | | | | | | |
|  | **B** | **CD14+ Mono** | **CD8+ T** | **DC** | **FCGR3A+ Mono** | **Memory CD4+** | **Naive CD4+ T** |
| **B** | 441 | 0 | 0 | 0 | 0 | 1 | 0 |
| **CD14+ Mono** | 0 | 1577 | 0 | 0 | 0 | 0 | 0 |
| **CD8+ T** | 0 | 0 | 225 | 0 | 0 | 0 | 0 |
| **DC** | 0 | 0 | 0 | 32 | 0 | 0 | 0 |
| **FCGR3A+ Mono** | 0 | 0 | 0 | 0 | 131 | 0 | 0 |
| **Memory CD4+** | 0 | 0 | 0 | 0 | 0 | 637 | 0 |
| **Naive CD4+ T** | 0 | 0 | 0 | 0 | 0 | 0 | 626 |
| **Enh 0.1 & Imp 0.75** | | | | | | | |
|  | **B** | **CD14+ Mono** | **CD8+ T** | **DC** | **FCGR3A+ Mono** | **Memory CD4+** | **Naive CD4+ T** |
| **B** | 442 | 0 | 0 | 0 | 0 | 0 | 0 |
| **CD14+ Mono** | 0 | 1577 | 0 | 0 | 0 | 0 | 0 |
| **CD8+ T** | 0 | 0 | 225 | 0 | 0 | 0 | 0 |
| **DC** | 0 | 0 | 0 | 32 | 0 | 0 | 0 |
| **FCGR3A+ Mono** | 0 | 0 | 0 | 0 | 131 | 0 | 0 |
| **Memory CD4+** | 0 | 0 | 0 | 0 | 0 | 637 | 0 |
| **Naive CD4+ T** | 0 | 0 | 0 | 0 | 0 | 0 | 626 |
| **Enh 0.3 & Imp 0.5** | | | | | | | |
|  | **B** | **CD14+ Mono** | **CD8+ T** | **DC** | **FCGR3A+ Mono** | **Memory CD4+** | **Naive CD4+ T** |
| **B** | 440 | 1 | 0 | 0 | 0 | 1 | 0 |
| **CD14+ Mono** | 0 | 1577 | 0 | 0 | 0 | 0 | 0 |
| **CD8+ T** | 0 | 1 | 214 | 0 | 0 | 0 | 10 |
| **DC** | 0 | 2 | 0 | 30 | 0 | 0 | 0 |
| **FCGR3A+ Mono** | 0 | 7 | 0 | 0 | 124 | 0 | 0 |
| **Memory CD4+** | 0 | 6 | 0 | 0 | 0 | 625 | 6 |
| **Naive CD4+ T** | 0 | 1 | 0 | 0 | 0 | 0 | 625 |
| **Enh 0.2 & Imp 0.5** | | | | | | | |
|  | **B** | **CD14+ Mono** | **CD8+ T** | **DC** | **FCGR3A+ Mono** | **Memory CD4+** | **Naive CD4+ T** |
| **B** | 442 | 0 | 0 | 0 | 0 | 0 | 0 |
| **CD14+ Mono** | 0 | 1577 | 0 | 0 | 0 | 0 | 0 |
| **CD8+ T** | 0 | 0 | 225 | 0 | 0 | 0 | 0 |
| **DC** | 0 | 0 | 0 | 32 | 0 | 0 | 0 |
| **FCGR3A+ Mono** | 0 | 0 | 0 | 0 | 131 | 0 | 0 |
| **Memory CD4+** | 0 | 0 | 0 | 0 | 0 | 637 | 0 |
| **Naive CD4+ T** | 0 | 0 | 0 | 0 | 0 | 0 | 626 |
| **Enh 0.1 & Imp 0.5** | | | | | | | |
|  | **B** | **CD14+ Mono** | **CD8+ T** | **DC** | **FCGR3A+ Mono** | **Memory CD4+** | **Naive CD4+ T** |
| **B** | 442 | 0 | 0 | 0 | 0 | 0 | 0 |
| **CD14+ Mono** | 0 | 1577 | 0 | 0 | 0 | 0 | 0 |
| **CD8+ T** | 0 | 0 | 225 | 0 | 0 | 0 | 0 |
| **DC** | 0 | 0 | 0 | 32 | 0 | 0 | 0 |
| **FCGR3A+ Mono** | 0 | 0 | 0 | 0 | 131 | 0 | 0 |
| **Memory CD4+** | 0 | 0 | 0 | 0 | 0 | 637 | 0 |
| **Naive CD4+ T** | 0 | 0 | 0 | 0 | 0 | 0 | 626 |
| **Enh 0.3 & Imp 0.25** | | | | | | | |
|  | **B** | **CD14+ Mono** | **CD8+ T** | **DC** | **FCGR3A+ Mono** | **Memory CD4+** | **Naive CD4+ T** |
| **B** | 441 | 0 | 0 | 0 | 0 | 0 | 1 |
| **CD14+ Mono** | 0 | 1577 | 0 | 0 | 0 | 0 | 0 |
| **CD8+ T** | 0 | 1 | 218 | 0 | 0 | 0 | 6 |
| **DC** | 0 | 2 | 0 | 30 | 0 | 0 | 0 |
| **FCGR3A+ Mono** | 0 | 4 | 0 | 0 | 127 | 0 | 0 |
| **Memory CD4+** | 0 | 6 | 0 | 0 | 0 | 626 | 5 |
| **Naive CD4+ T** | 0 | 1 | 0 | 0 | 0 | 0 | 625 |
| **Enh 0.2 & Imp 0.25** | | | | | | | |
|  | **B** | **CD14+ Mono** | **CD8+ T** | **DC** | **FCGR3A+ Mono** | **Memory CD4+** | **Naive CD4+ T** |
| **B** | 442 | 0 | 0 | 0 | 0 | 0 | 0 |
| **CD14+ Mono** | 0 | 1577 | 0 | 0 | 0 | 0 | 0 |
| **CD8+ T** | 0 | 0 | 225 | 0 | 0 | 0 | 0 |
| **DC** | 0 | 0 | 0 | 32 | 0 | 0 | 0 |
| **FCGR3A+ Mono** | 0 | 0 | 0 | 0 | 131 | 0 | 0 |
| **Memory CD4+** | 0 | 0 | 0 | 0 | 0 | 637 | 0 |
| **Naive CD4+ T** | 0 | 0 | 0 | 0 | 0 | 0 | 626 |
| **Enh 0.1 & Imp 0.25** | | | | | | | |
|  | **B** | **CD14+ Mono** | **CD8+ T** | **DC** | **FCGR3A+ Mono** | **Memory CD4+** | **Naive CD4+ T** |
| **B** | 442 | 0 | 0 | 0 | 0 | 0 | 0 |
| **CD14+ Mono** | 0 | 1577 | 0 | 0 | 0 | 0 | 0 |
| **CD8+ T** | 0 | 0 | 225 | 0 | 0 | 0 | 0 |
| **DC** | 0 | 0 | 0 | 32 | 0 | 0 | 0 |
| **FCGR3A+ Mono** | 0 | 0 | 0 | 0 | 131 | 0 | 0 |
| **Memory CD4+** | 0 | 0 | 0 | 0 | 0 | 637 | 0 |
| **Naive CD4+ T** | 0 | 0 | 0 | 0 | 0 | 0 | 626 |

*Note*: In each table, the row represents the true label of cells and column represents the predicted label of cells
