# Supplementary material for: Enhancement and Imputation of Peak Signal Enables Accurate Cell-Type Classification in scATAC-seq
Source: Front Genet. 2021 Apr 6;12:658352. doi: 10.3389/fgene.2021.658352 (PMC8056015; doi:10.3389/fgene.2021.658352)
Supplement: Supplementary Table 9 — F1 scores of inter-dataset experiment that training with 10× PBMCs v1 Seurat Labeled dataset and predicting in 10× PBMCs Next Gem Seurat Labeled dataset with different enhancement and imputation cutoffs. [file Table_9.DOCX]

**Supplementary Table 9 F1 scores of inter-dataset experiment that training with 10x PBMCs v1 Seurat Labelled dataset and predicting in 10x PBMCs Next Gem Seurat Labelled dataset with different enhancement and imputation cutoffs**

| **F1 score** | **B** | **CD14+ Mono** | **CD8+ T** | **DC** | **FCGR3A+ Mono** | **Memory CD4+** | **Naive CD4+ T** |
| --- | --- | --- | --- | --- | --- | --- | --- |
| No Enhancement & No Imputation | 0.9931973 | 0.952237 | 0 | 0.6086957 | 0.04477612 | 0.90338563 | 0.78617992 |
| Enh 0.3 & No Imp | 0 | 0.59302326 | 0 | 0 | 0 | 0 | 0.02836879 |
| Enh 0.3 & Imp 0.75 | 0.9943117 | 0.95338983 | 0 | 0.3589744 | 0.21768707 | 0.91578087 | 0.79448457 |
| Enh 0.3 & Imp 0.5 | 0.9931663 | 0.94765343 | 0 | 0 | 0.04477612 | 0.91578087 | 0.79527559 |
| Enh 0.3 & Imp 0.25 | 0.9897143 | 0.94594595 | 0 | 0 | 0 | 0.92282697 | 0.7994723 |
| Enh 0.2 & No Imp | 0 | 0.59475219 | 0 | 0 | 0 | 0.01221374 | 0.03098592 |
| Enh 0.2 & Imp 0.75 | 0.9920182 | 0.94629463 | 0 | 0 | 0 | 0.97266881 | 0.83444593 |
| Enh 0.2 & Imp 0.5 | 0.9897143 | 0.9460108 | 0 | 0 | 0 | 0.97998399 | 0.83668005 |
| Enh 0.2 & Imp 0.25 | 0.9815668 | 0.94402873 | 0 | 0 | 0 | 0.97008892 | 0.83001328 |
| Enh 0.1 & No Imp | 0.0950966 | 0.47195013 | 0 | 0 | 0 | 0.08244681 | 0.25208526 |
| Enh 0.1 & Imp 0.75 | 1 | 1 | 0 | 0.98412698 | 1 | 1 | 0.847090663 |
| Enh 0.1 & Imp 0.5 | 1 | 1 | 0 | 1 | 1 | 1 | 0.84766418 |
| Enh 0.1 & Imp 0.25 | 1 | 0.00758054 | 1 | 1 | 0.14293508 | 1 | 1 |
