# Supplementary material for: Enhancement and Imputation of Peak Signal Enables Accurate Cell-Type Classification in scATAC-seq
Source: Front Genet. 2021 Apr 6;12:658352. doi: 10.3389/fgene.2021.658352 (PMC8056015; doi:10.3389/fgene.2021.658352)
Supplement: Supplementary Table 10 — The confusion matrix across different enhancement and imputation cutoffs for inter-dataset experiment that training with 10× PBMCs v1 Seurat Labeled dataset and predicting in 10× PBMCs Next Gem Seurat Labeled dataset. [file Table_10.DOCX]

**Supplementary Table 10 The confusion matrix across different enhancement and imputation cutoffs for inter-dataset experiment that training with 10x PBMCs v1 Seurat Labelled dataset and predicting in 10x PBMCs Next Gem Seurat Labelled dataset**

| **No Enh & No Imp** | | | | | | | |
| --- | --- | --- | --- | --- | --- | --- | --- |
|  | **B** | **CD14+ Mono** | **CD8+ T** | **DC** | **FCGR3A+ Mono** | **Memory CD4+** | **Naive CD4+ T** |
| **B** | 438 | 2 | 0 | 0 | 0 | 2 | 0 |
| **CD14+ Mono** | 0 | 1575 | 0 | 0 | 0 | 2 | 0 |
| **CD8+ T** | 0 | 1 | 0 | 0 | 0 | 2 | 222 |
| **DC** | 1 | 16 | 0 | 14 | 0 | 1 | 0 |
| **FCGR3A+ Mono** | 0 | 128 | 0 | 0 | 3 | 0 | 0 |
| **Memory CD4+** | 0 | 7 | 0 | 0 | 0 | 547 | 83 |
| **Naive CD4+ T** | 1 | 2 | 0 | 0 | 0 | 20 | 603 |
| **Enh 0.3 & No Imp** | | | | | | | |
|  | **B** | **CD14+ Mono** | **CD8+ T** | **DC** | **FCGR3A+ Mono** | **Memory CD4+** | **Naive CD4+ T** |
| **B** | 0 | 433 | 0 | 0 | 0 | 1 | 8 |
| **CD14+ Mono** | 0 | 1530 | 0 | 0 | 0 | 6 | 41 |
| **CD8+ T** | 0 | 218 | 0 | 0 | 0 | 1 | 6 |
| **DC** | 0 | 30 | 0 | 0 | 0 | 0 | 2 |
| **FCGR3A+ Mono** | 0 | 128 | 0 | 0 | 0 | 0 | 3 |
| **Memory CD4+** | 0 | 628 | 0 | 0 | 0 | 0 | 9 |
| **Naive CD4+ T** | 0 | 616 | 0 | 0 | 0 | 0 | 10 |
| **Enh 0.2 & No Imp** | | | | | | | |
|  | **B** | **CD14+ Mono** | **CD8+ T** | **DC** | **FCGR3A+ Mono** | **Memory CD4+** | **Naive CD4+ T** |
| **B** | 0 | 433 | 0 | 0 | 0 | 1 | 8 |
| **CD14+ Mono** | 0 | 1530 | 0 | 0 | 0 | 9 | 38 |
| **CD8+ T** | 0 | 219 | 0 | 0 | 0 | 2 | 4 |
| **DC** | 0 | 32 | 0 | 0 | 0 | 0 | 0 |
| **FCGR3A+ Mono** | 0 | 127 | 0 | 0 | 0 | 0 | 4 |
| **Memory CD4+** | 0 | 614 | 0 | 0 | 0 | 4 | 19 |
| **Naive CD4+ T** | 0 | 613 | 0 | 0 | 0 | 2 | 11 |
| **Enh 0.1 & No Imp** | | | | | | | |
|  | **B** | **CD14+ Mono** | **CD8+ T** | **DC** | **FCGR3A+ Mono** | **Memory CD4+** | **Naive CD4+ T** |
| **B** | 32 | 215 | 0 | 0 | 0 | 9 | 186 |
| **CD14+ Mono** | 107 | 795 | 0 | 0 | 0 | 40 | 635 |
| **CD8+ T** | 13 | 109 | 0 | 0 | 0 | 5 | 98 |
| **DC** | 2 | 16 | 0 | 0 | 0 | 1 | 13 |
| **FCGR3A+ Mono** | 3 | 52 | 0 | 0 | 0 | 9 | 67 |
| **Memory CD4+** | 39 | 306 | 0 | 0 | 0 | 31 | 261 |
| **Naive CD4+ T** | 35 | 299 | 0 | 0 | 0 | 20 | 272 |
| **Enh 0.3 & Imp 0.75** | | | | | | | |
|  | **B** | **CD14+ Mono** | **CD8+ T** | **DC** | **FCGR3A+ Mono** | **Memory CD4+** | **Naive CD4+ T** |
| **B** | 437 | 2 | 0 | 0 | 0 | 3 | 0 |
| **CD14+ Mono** | 0 | 1575 | 0 | 0 | 0 | 2 | 0 |
| **CD8+ T** | 0 | 1 | 0 | 0 | 0 | 2 | 222 |
| **DC** | 0 | 25 | 0 | 7 | 0 | 0 | 0 |
| **FCGR3A+ Mono** | 0 | 115 | 0 | 0 | 16 | 0 | 0 |
| **Memory CD4+** | 0 | 7 | 0 | 0 | 0 | 560 | 70 |
| **Naive CD4+ T** | 0 | 2 | 0 | 0 | 0 | 19 | 605 |
| **Enh 0.2 & Imp 0.75** | | | | | | | |
|  | **B** | **CD14+ Mono** | **CD8+ T** | **DC** | **FCGR3A+ Mono** | **Memory CD4+** | **Naive CD4+ T** |
| **B** | 435 | 5 | 0 | 0 | 0 | 2 | 0 |
| **CD14+ Mono** | 0 | 1577 | 0 | 0 | 0 | 0 | 0 |
| **CD8+ T** | 0 | 1 | 0 | 0 | 0 | 0 | 224 |
| **DC** | 0 | 32 | 0 | 0 | 0 | 0 | 0 |
| **FCGR3A+ Mono** | 0 | 131 | 0 | 0 | 0 | 0 | 0 |
| **Memory CD4+** | 0 | 9 | 0 | 0 | 0 | 605 | 23 |
| **Naive CD4+ T** | 0 | 1 | 0 | 0 | 0 | 0 | 625 |
| **Enh 0.1 & Imp 0.75** | | | | | | | |
|  | **B** | **CD14+ Mono** | **CD8+ T** | **DC** | **FCGR3A+ Mono** | **Memory CD4+** | **Naive CD4+ T** |
| **B** | 442 | 0 | 0 | 0 | 0 | 0 | 0 |
| **CD14+ Mono** | 0 | 1577 | 0 | 0 | 0 | 0 | 0 |
| **CD8+ T** | 0 | 0 | 0 | 0 | 0 | 0 | 225 |
| **DC** | 0 | 0 | 0 | 31 | 0 | 0 | 1 |
| **FCGR3A+ Mono** | 0 | 0 | 0 | 0 | 131 | 0 | 0 |
| **Memory CD4+** | 0 | 0 | 0 | 0 | 0 | 637 | 0 |
| **Naive CD4+ T** | 0 | 0 | 0 | 0 | 0 | 0 | 626 |
| **Enh 0.3 & Imp 0.5** | | | | | | | |
|  | **B** | **CD14+ Mono** | **CD8+ T** | **DC** | **FCGR3A+ Mono** | **Memory CD4+** | **Naive CD4+ T** |
| **B** | 436 | 2 | 0 | 0 | 0 | 4 | 0 |
| **CD14+ Mono** | 0 | 1575 | 0 | 0 | 0 | 2 | 0 |
| **CD8+ T** | 0 | 1 | 0 | 0 | 0 | 2 | 222 |
| **DC** | 0 | 32 | 0 | 7 | 0 | 0 | 0 |
| **FCGR3A+ Mono** | 0 | 128 | 0 | 0 | 3 | 0 | 0 |
| **Memory CD4+** | 0 | 7 | 0 | 0 | 0 | 560 | 70 |
| **Naive CD4+ T** | 0 | 2 | 0 | 0 | 0 | 18 | 606 |
| **Enh 0.2 & Imp 0.5** | | | | | | | |
|  | **B** | **CD14+ Mono** | **CD8+ T** | **DC** | **FCGR3A+ Mono** | **Memory CD4+** | **Naive CD4+ T** |
| **B** | 433 | 6 | 0 | 0 | 0 | 0 | 3 |
| **CD14+ Mono** | 0 | 1577 | 0 | 0 | 0 | 0 | 0 |
| **CD8+ T** | 0 | 1 | 0 | 0 | 0 | 0 | 224 |
| **DC** | 0 | 32 | 0 | 0 | 0 | 0 | 0 |
| **FCGR3A+ Mono** | 0 | 131 | 0 | 0 | 0 | 0 | 0 |
| **Memory CD4+** | 0 | 9 | 0 | 0 | 0 | 612 | 16 |
| **Naive CD4+ T** | 0 | 1 | 0 | 0 | 0 | 0 | 625 |
| **Enh 0.1 & Imp 0.5** | | | | | | | |
|  | **B** | **CD14+ Mono** | **CD8+ T** | **DC** | **FCGR3A+ Mono** | **Memory CD4+** | **Naive CD4+ T** |
| **B** | 442 | 0 | 0 | 0 | 0 | 0 | 0 |
| **CD14+ Mono** | 0 | 1577 | 0 | 0 | 0 | 0 | 0 |
| **CD8+ T** | 0 | 0 | 0 | 0 | 0 | 0 | 225 |
| **DC** | 0 | 0 | 0 | 32 | 0 | 0 | 0 |
| **FCGR3A+ Mono** | 0 | 0 | 0 | 0 | 131 | 0 | 0 |
| **Memory CD4+** | 0 | 0 | 0 | 0 | 0 | 637 | 0 |
| **Naive CD4+ T** | 0 | 0 | 0 | 0 | 0 | 0 | 626 |
| **Enh 0.3 & Imp 0.25** | | | | | | | |
|  | **B** | **CD14+ Mono** | **CD8+ T** | **DC** | **FCGR3A+ Mono** | **Memory CD4+** | **Naive CD4+ T** |
| **B** | 433 | 5 | 0 | 0 | 0 | 4 | 0 |
| **CD14+ Mono** | 0 | 1575 | 0 | 0 | 0 | 2 | 0 |
| **CD8+ T** | 0 | 1 | 0 | 0 | 0 | 2 | 222 |
| **DC** | 0 | 32 | 0 | 7 | 0 | 0 | 0 |
| **FCGR3A+ Mono** | 0 | 131 | 0 | 0 | 3 | 0 | 0 |
| **Memory CD4+** | 0 | 7 | 0 | 0 | 0 | 568 | 62 |
| **Naive CD4+ T** | 0 | 2 | 0 | 0 | 0 | 18 | 606 |
| **Enh 0.2 & Imp 0.25** | | | | | | | |
|  | **B** | **CD14+ Mono** | **CD8+ T** | **DC** | **FCGR3A+ Mono** | **Memory CD4+** | **Naive CD4+ T** |
| **B** | 426 | 13 | 0 | 0 | 0 | 0 | 3 |
| **CD14+ Mono** | 0 | 1577 | 0 | 0 | 0 | 0 | 0 |
| **CD8+ T** | 0 | 1 | 0 | 0 | 0 | 0 | 224 |
| **DC** | 0 | 32 | 0 | 0 | 0 | 0 | 0 |
| **FCGR3A+ Mono** | 0 | 131 | 0 | 0 | 0 | 0 | 0 |
| **Memory CD4+** | 0 | 9 | 0 | 0 | 0 | 600 | 28 |
| **Naive CD4+ T** | 0 | 1 | 0 | 0 | 0 | 0 | 625 |
| **Enh 0.1 & Imp 0.25** | | | | | | | |
|  | **B** | **CD14+ Mono** | **CD8+ T** | **DC** | **FCGR3A+ Mono** | **Memory CD4+** | **Naive CD4+ T** |
| **B** | 442 | 0 | 0 | 0 | 0 | 0 | 0 |
| **CD14+ Mono** | 0 | 6 | 0 | 0 | 1571 | 0 | 0 |
| **CD8+ T** | 0 | 0 | 225 | 0 | 0 | 0 | 0 |
| **DC** | 0 | 0 | 0 | 32 | 0 | 0 | 0 |
| **FCGR3A+ Mono** | 0 | 0 | 0 | 0 | 131 | 0 | 0 |
| **Memory CD4+** | 0 | 0 | 0 | 0 | 0 | 637 | 0 |
| **Naive CD4+ T** | 0 | 0 | 0 | 0 | 0 | 0 | 626 |

*Note*: In each table, the row represents the true label of cells and column represents the predicted label of cells
